# Active Biopaste for Coral Reef Restoration

**DOI:** 10.1002/adma.202502078

**Published:** 2025-07-04

**Authors:** Gabriele Corigliano, Valerio Isa, Valerio Francesco Annese, Camilla Rinaldi, Maria Summa, Rosalia Bertorelli, Paolo Galli, Silvia Lavorano, Mario Caironi, Marco Contardi, Pietro Cataldi, Simone Montano, Athanassia Athanassiou

**Affiliations:** ^1^ Smart Materials Istituto Italiano di Tecnologia Via Morego 30 Genova 16163 Italy; ^2^ Department of Earth and Environmental Sciences (DISAT) University of Milano – Bicocca Piazza della Scienza Milan 20126 Italy; ^3^ MaRHE Center (Marine Research and High Education Center) Magoodhoo Island Faafu Atoll 12030 Republic of Maldives; ^4^ Center for Nano Science and Technology Istituto Italiano di Tecnologia Via Rubattino 81 Milano 20134 Italy; ^5^ Translational Pharmacology Istituto Italiano di Tecnologia Via Morego 30 Genova 16163 Italy; ^6^ Dubai Business School University of Dubai Dubai 14143 United Arab Emirates; ^7^ Costa Edutainment SpA – Acquario di Genova Ponte Spinola Genova 16128 Italy

**Keywords:** biocomposite, coral reefs, coral restoration, green electronics, mineral accretion, soybean oil, underwater

## Abstract

Preserving coral reefs is crucial for safeguarding marine biodiversity, global ecosystems, and coastal communities. Coral restoration focuses on farming and transplanting corals back onto reefs. However, traditional attachment methods, such as petroleum‐based epoxy, pose environmental risks or provide inefficient affixation. Moreover, maximizing coral growth while farming boosts the restoration rate of the reefs. Hence, an environmentally friendly, conductive hardening bicomponent paste is developed to transplant and anchor corals, provide them with a solid growing substrate, and enable mineral accretion technology (MAT), a strategy to accelerate coral farming. The bicomponent paste consists of bio‐based and biodegradable acrylate soybean oil matrix and graphene nanoplatelets fillers. The paste hardens through mixing, transitioning from a Young's modulus of ≃0.1 to ≃60 MPa and reaching a strength of ≃5 MPa. Rheological tests demonstrate the tunability of the crosslinking dynamics of the paste. The paste exhibits a resistivity of 0.1 Ω∙m, with stable electrical properties for over 40 days in seawater. MAT tests show significant enhancement of coral growth rates within two weeks, doubling those of the control group. This paste offers versatility for application in aquaria and nurseries, does not require prone‐to‐oxidation metallic structures underwater, and can be employed on reefs.

## Graphical abstract

Preserving coral reefs is essential. A conductive, hardening paste for coral attachment and mineral accretion technology is developed, using a bio‐based acrylate soybean oil matrix and graphene nanoplatelets. Corals are attached with and onto the conductive paste, electricity is applied, water electrolysis is induced, and calcium is deposited at the cathode to enhance coral growth via faster calcium carbonate deposition.

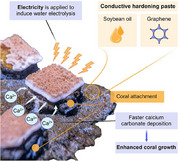

## 1 Introduction

Coral reef ecosystems sustain 25% of global marine biodiversity,^[^ ^1^ ^]^ are estimated to provide nourishment to hundreds of millions of people, and benefit one billion individuals ^[^ ^2^, ^3^, ^4^ ^]^ with a societal value ≈€350 billion annually. However, these ecosystems face swift degradation due to climate change, ocean acidification, and destructive fishing practices.^[^ ^5^, ^6^ ^]^ In recent years, there has been a reduction of at least 20% in the amount of coral reefs present globally, with some assessments indicating losses as high as 50%.^[^ ^7^ ^]^ Projections suggest a further decline of 70%–90% by 2050 if ocean temperatures rise by 1.5 °C,^[^ ^2^, ^7^ ^]^ and a risk of total extinction if temperatures increase by 2.0°C. For these reasons, advancing work aimed at preserving coral reefs is of the utmost importance.

Coral reef restoration has thus become a topic of increasing global interest, with a multitude of strategies being explored in an effort to rejuvenate and safeguard coral ecosystems.^[^ ^8^, ^9^, ^10^ ^]^ Approximately 20% of restoration projects involve direct coral transplantation from donor reefs, 5% entail microfragmentation of slow‐growing corals, and ≈50% utilize the “coral‐gardening” approach.^[^ ^9^ ^]^ The coral gardening method entails farming coral fragments under optimal conditions in artificial nurseries, either in situ or ex situ (in aquaria), before their transplantation onto designated restoration sites within a reef.^[^ ^11^, ^12^, ^13^, ^14^, ^15^ ^]^ Coral gardening has an average coral survival rate of 66%,^[^ ^9^ ^]^ which can vary as a function of species, techniques utilized for their transplant, and the influence of their surrounding environment. Successful coral gardening depends on several environmental parameters (e.g., water temperature,^[^ ^17^ ^]^ pH,^[^ ^18^ ^]^ water quality,^[^ ^19^ ^]^ lighting conditions,^[^ ^20^ ^]^ salinity,^[^ ^21^ ^]^ and nutrient levels)^[^ ^22^ ^]^ that enable the colonization of coralline algae and promote coral settlement.^[^ ^16^ ^]^

Another essential aspect of coral transplantation is ensuring that coral fragments' attachment to substrates, both in nurseries and onto the reef, is long‐lasting and efficient. Corals have been demonstrated to thrive when firmly anchored to a hard substrate, but they struggle to survive if placed on a soft surface or left unanchored.^[^ ^23^, ^24^ ^]^ Coral attachment is commonly facilitated using hardening bicomponent pastes,^[^ ^25^, ^26^, ^27^ ^]^ coral glues, cable ties, coral clips, metallic wires, nails, and marine concretes.^[^ ^27^, ^28^, ^29^ ^]^ Epoxy putties are paste‐like, malleable, two‐component materials that solidify upon mixing. Although widely used in coral restoration, they were not specifically designed for this purpose. Being non‐biobased and non‐biodegradable, they contribute to persistent plastic waste accumulation if used for coral reef restoration^[^ ^30^ ^]^ and potentially leach chemicals that are toxic to marine organisms.^[^ ^31^, ^32^ ^]^ Cyanoacrylate gel adhesives cure quickly but are difficult to handle underwater, have poor mechanical strength, and are prone to failure, limiting their use to small coral fragments in reduced current conditions.^[^ ^33^ ^]^ Biodegradable UV‐photocurable glues offer strong bonding but require UV light to cure, which complicates their use in the field.^[^ ^34^ ^]^ Wires, cables, clips, and nails are rapid attachment methods; however, they do not guarantee a secure bond once the coral fragment is positioned. Marine concretes are not suitable for manual handling,^[^ ^35^ ^]^ have to be mixed out of the water, and typically require specific tools like an appropriate gun or piping bag for positioning.^[^ ^28^ ^]^ On top of these complications, they have been proven to induce tissue degradation in polyps’ micropropagation.^[^ ^35^ ^]^ This results in lower accuracy, reliability, and safety during coral transplantation compared to bicomponent pastes. Additionally, the extended hardening times of commercial epoxy putties and concretes may lead to inadequate adhesion of transplanted corals to reefs, increasing vulnerability to detachment due to ocean currents.^[^ ^33^ ^]^

Coral growth rates are often slow. In literature, monthly growth rates between 0.3 and 4 mm are reported for species of stony corals such as *Pavona spp*, *Pocillopora spp*., *Montastrea spp*., *Porites spp*., *Favia spp*., *Goniastrea spp*., and *Psammocora spp*.^[^ ^36^, ^37^, ^38^ ^]^ Thus, optimizing coral restoration techniques to improve coral growth rates and recovery outcomes would provide a significant benefit to reef ecosystems. One effective method employed to facilitate growth rate improvements is Biorock technology, also known as mineral accretion technology (MAT).^[^ ^39^, ^40^, ^41^, ^42^, ^43^, ^44^ ^]^ This technique boosts coral growth through mineral accretion driven by electrochemical processes. MAT exploits a direct electrical current between two electrodes immersed in an electrolyte, like seawater, to promote water electrolysis; precipitating and agglomerating calcium carbonates, magnesium hydroxides, and hydrogen at the cathode while oxygen and chlorine are produced at the anode. Anchoring corals at the cathode accelerates the deposition rate of their calcium carbonate matrix, increasing resilience against stress factors.

Although effective in creating new healthy reefs, this solution entails complex placement and lacks versatility. In a standard MAT setup, corals are attached to a large metallic framework, spanning several square meters, which functions as the cathode. Electrical current is supplied underwater through cables connected to a nearby shore‐based power source, and the setup functions effectively as long as power is supplied. When the power supply is interrupted, the protective layer grown on the metallic frame through mineral accretion disaggregates. Subsequent exposure of the frame to seawater leads to metal oxidation and rust formation, which can release toxic ions harmful to corals and other marine organisms.^[^ ^45^, ^46^, ^47^ ^]^ Furthermore, expert operators, such as marine biologists, must attach corals to the metallic frame to guarantee a correct attachment using the abovementioned materials and tools.

Considering all these factors, integrating efficient coral attachment with rust‐free electrochemical mineral accretion would be an effective tool to enhance coral growth practically and sustainably. A potential pathway for achieving this goal is the development of a green, electrically conductive paste or adhesive that could simultaneously facilitate coral attachment and act as an electrode for MAT. Additionally, this paste, or adhesive, should be carefully designed to be capable of maintaining stable properties in underwater environments.

The literature already provides examples of conductive adhesives and pastes, including those formulated with durable matrices such as epoxy,^[^ ^48^, ^49^ ^]^ as well as conductive mortars and concretes employed in construction as sensors.^[^ ^50^, ^51^, ^52^, ^53^, ^54^, ^55^, ^56^, ^57^, ^58^, ^59^, ^60^, ^61^, ^62^, ^63^, ^64^ ^]^ These solutions are associated with the previously discussed drawbacks linked to petroleum‐derived, long‐lasting epoxy and difficult‐to‐handle concrete and were not specifically designed for employment underwater. Conductive biopastes have been developed using conductive polymers, including PEDOT:PSS, polypyrrole, polyaniline, and melanin,^[^ ^65^ ^]^ integrated into various biodegradable matrices for biomedical applications. PEDOT:PSS has been combined with cellulose,^[^ ^66^ ^]^ chitosan, and starch,^[^ ^67^ ^]^ carrageenan, and PDA‐PAM networks,^[^ ^68^ ^]^ as well as with carbon fillers and cellulose.^[^ ^69^ ^]^ Polypyrrole has been paired with alginate,^[^ ^70^ ^]^ cellulose,^[^ ^71^, ^72^ ^]^ collagen,^[^ ^73^ ^]^ silk and tannic acid.^[^ ^74^ ^]^ Polyaniline is often used in biosensing alongside metallic^[^ ^75^ ^]^ and carbon fillers^[^ ^76^, ^77^ ^]^ or both,**^[^** ^78^, ^79^ ^]^ combined with natural matrices like chitosan,**^[^** ^75^ **^,^** ^78^ **^]^** ethylcellulose,**^[^** ^78^ **^]^** and starch.**^[^** ^76^, ^77^ ^]^ Melanin has been employed for on‐skin electronics,^[^ ^80^, ^81^, ^82^ ^]^ however, despite being highly biodegradable, it is primarily an ionic conductor with limited electronic conductivity. Furthermore, all of the biopastes listed above, in most cases, are not amenable to adaptation to coral reef restoration as they lack hardness and adhesive properties, degrade too fast, or are not stable underwater.

Conductive biopastes have also been formulated using biobased, biodegradable, or bioresorbable binding materials, incorporating both metallic and carbon‐based fillers. Bioresorbable and biodegradable conductive biopastes have been developed for medical applications using natural waxes or biodegradable polymers with transient metallic fillers like tungsten powder,^[^ ^83^, ^84^, ^85^ ^]^ molybdenum,^[^ ^86^, ^87^ ^]^ and zinc.^[^ ^88^, ^89^, ^90^, ^91^ ^]^ Metals offer excellent conductivity, but are expensive and prone to corrosion, oxidation, or dissolution in water‐based media, depending on their properties.^[^ ^92^, ^93^, ^94^ ^]^ On the other hand, carbon‐based fillers such as activated carbon, graphene‐related materials, carbon black, reduced graphene oxide, and carbon nanotubes and nanofibers, are not easily corroded and oxidized compared to metallic ones, are more cost‐effective, exhibit better stability underwater, are easier to blend into composites, and offer higher tunability of electrical properties based on the type and amount of filler used.^[^ ^95^, ^96^, ^97^ ^]^ As such, conductive carbon biopastes are widely utilized in a variety of flexible and printed electronic devices, spanning from wearable electronics^[^ ^95^, ^98^ ^]^ to optoelectronics.^[^ ^99^, ^100^, ^101^ ^]^ Conductive carbon biopastes have been developed using beeswax,^[^ ^102^, ^103^ ^]^ methylcellulose,^[^ ^104^, ^105^, ^106^ ^]^ starch,^[^ ^107^, ^108^, ^109^ ^]^ fruit waste^[^ ^110^ ^]^ or PLGA.^[^ ^111^ ^]^ However, these materials exhibit limitations such as rapid degradation, hygroscopicity, high water solubility, and low mechanical stiffness, making them inappropriate for underwater applications as substrates for coral growth. Conductive carbon biopastes, composed of beeswax, sunflower oil, zein, and activated carbon, were formulated to be edible.^[^ ^112^, ^113^ ^]^ While these solutions exhibit excellent biocompatibility and stability of the electrical properties underwater, they are not hardening pastes, and their softness renders them unsuitable as a substrate for coral anchoring and growth.

Among all formulations discussed so far, there is the lack of a malleable, green conductive paste that can be molded underwater accurately by hand, can harden with tunable curing times and on request, can maintain consistent adhesive properties and stability for prolonged periods in seawater, and can sustain a constant voltage underwater for weeks to facilitate application of MAT.

In this article, a bicomponent conductive hardening biopaste specifically designed to facilitate the rapid, eco‐friendly transplantation of corals and to enhance the deposition of their calcium carbonate matrix via electrochemically‐driven mineral accretion is introduced. The paste is composed of a bio‐based and biodegradable matrix and a conductive carbon‐based filler. The selected green matrix is epoxidized soybean oil acrylate (ESOA), while graphene nanoplatelets (GnPs) are the employed conductive fillers. One component of the biopaste is loaded with a radical initiator, while the other is loaded with an accelerator. Mixing the two ingredients crosslinks the polymeric chains and hence hardens the oily matrix.^[^ ^114^ ^]^ This green solution offers versatility for applications in ex situ and in situ nurseries, does not require long‐lasting metallic structures underwater, and can potentially be directly applied to the reef.

## 2 Results and Discussion

The matrix of the conductive hardening paste consists of ESOA, a bio‐based and renewable alternative to petrochemicals in polymer science.^[^ ^115^ ^]^ ESOA, known for being non‐toxic and having high chemical reactivity,^[^ ^116^ ^]^ is derived from soybean oil, a widely available and inexpensive feedstock.^[^ ^117^ ^]^ As electrically conductive fillers, GnPs were selected. GnPs are a commercially available graphitic material with properties in between single‐layer graphene and graphite, consisting of nanoflakes with micrometric lateral dimensions and nanometric thickness.^[^ ^118^ ^]^ GnPs were considered the optimum carbon‐based filler for this material because they provide a balanced combination of high electrical conductivity, good mechanical and barrier properties, ease of processing, and cost‐effectiveness compared to tube‐like, spherical, or oxidized carbon fillers.^[^ ^119^, ^120^ ^]^ These qualities make GnPs potentially well‐suited for various underwater applications. Additionally, when mixed with ESOA, GnPs demonstrated better processability than other carbon‐based fillers, such as carbon nanofibers and activated carbon, allowing for higher filler loadings without sacrificing paste cohesiveness.

**Figure 1:**
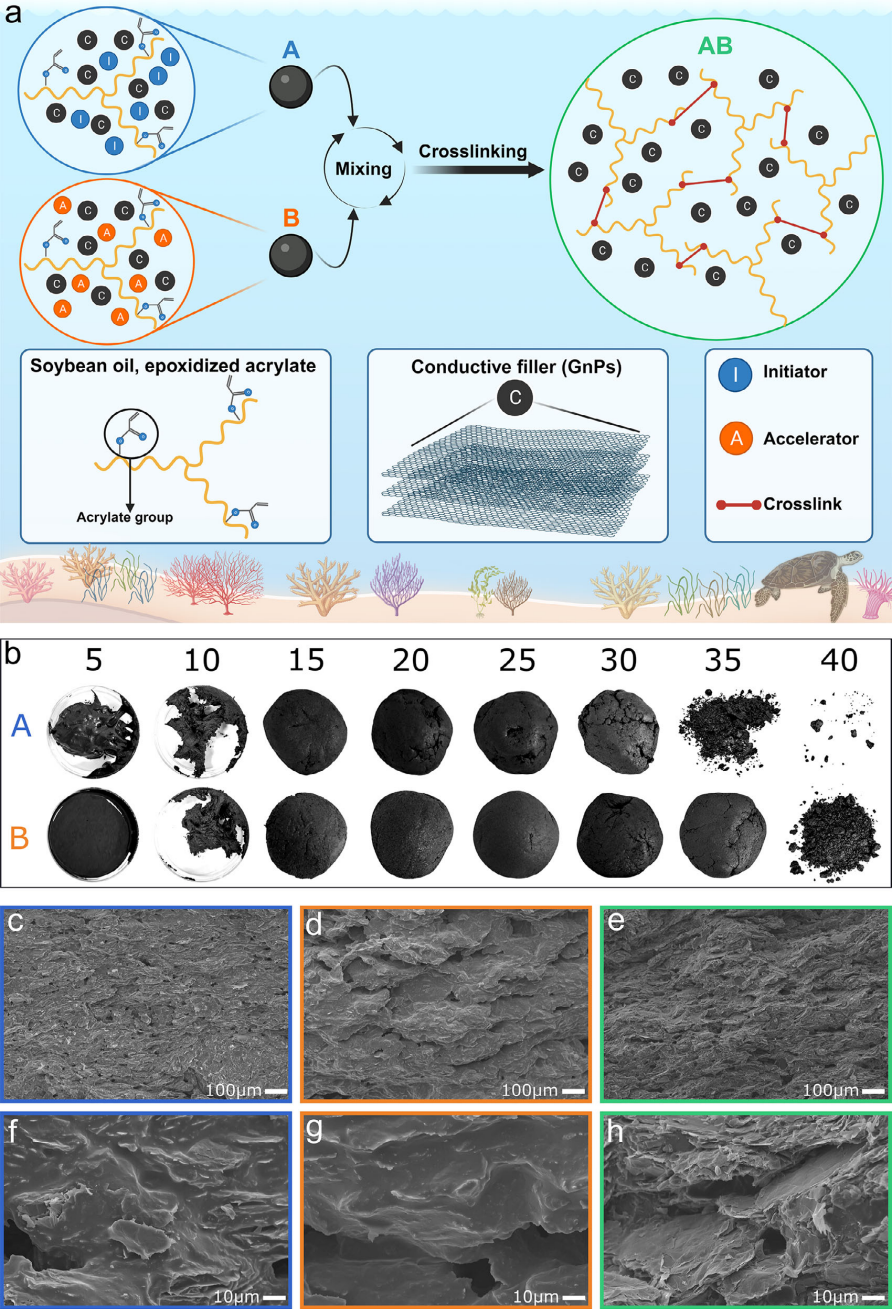
a) A schematic of the raw ingredients of the bicomponent conductive paste, named A and B before mixing, and its crosslinking mechanism. A and B are made with ESOA, GnPs, and a solid initiator or a liquid accelerator, respectively. The crosslinked paste is named AB. b) Photos of different preparations of conductive components as a function of GnPs loading (in wt.%). c–h) SEM images of cross‐sections of components A (blue) and B (orange), as well as paste AB (green) at different magnifications. All the presented SEM images display pastes with a GnPs loading of 25 wt.%.

As shown in the schematic of **Figure** **1**a, the paste has two components, labelled A and B, which both contain ESOA and GnPs. Component A additionally contains the solid initiator lauroyl peroxide, while component B contains the liquid accelerator para‐toluidine ethoxylate tertiary amine (known commercially as Bisomer PTE). When the two components are mixed, a radical reaction is triggered, and a crosslinking of the matrix occurs. The mixed paste, both as it is actively crosslinking and after it has completed the crosslinking process, is referred to as paste AB. To evaluate the pastes' properties, various formulations of components A and B were systematically characterized, as well as the resulting crosslinked AB pastes. First, the crosslinking reaction was confirmed through attenuated total reflection‐Fourier transform infrared (ATR‐FTIR) spectroscopy. Spectra of components A and B, paste AB, and a control of pure ESOA were collected for comparison (Figures S1 and S2, Supporting Information). The peak corresponding to the acrylate group, observed at 1637 cm^−1^, has a significantly diminished intensity in the AB paste compared to the individual components alone, which indicates successful crosslinking.^[^ ^114^ ^]^

Once successful crosslinking was confirmed, further work was done to understand the influence that the composition of the individual components would have on the final properties obtained. In Figure 1b, photographs of components A and B with GnPs loading concentrations ranging from 5 to 40 wt.%. are shown. The percentage is calculated as the weight of GnPs divided by the total weight of the paste. It can be observed that the components change from a liquid to a paste‐like consistency to a powdery material with no cohesion as the loading of GnPs increases. The highest percentages of GnPs significantly hampered the preparation of the AB paste by interfering with the homogenous mixing of components A and B, which inhibited the subsequent crosslinking reaction. The consistency also differs between component A and component B, with the former exhibiting greater brittleness and lower cohesion than the latter. This difference is likely induced by the incorporation of a solid initiator in component A. Figure 1c–h shows the micromorphology, through representative scanning electron microscopy (SEM) images of cross‐sections of components A (Figure 1c,f) and B (Figure 1d–g) with a GnPs loading of 25 wt.% and the corresponding AB paste they formed (Figure 1e,h). No clusters of lauroyl peroxide were detected in component A, highlighting the good miscibility between the solid initiator and the ESOA matrix. GnPs are distinguishable in every image, and their distribution appears consistently homogenous in components A and B. Furthermore, crosslinking does not seem to influence the distribution of GnPs throughout the ESOA since the nanoflakes appear homogeneously dispersed throughout the matrix in the AB paste samples. The interface between the matrix and the filler seems free from any gaps or voids, confirming good incorporation of the GnPs in the paste and its starting components.

**Figure 2:**
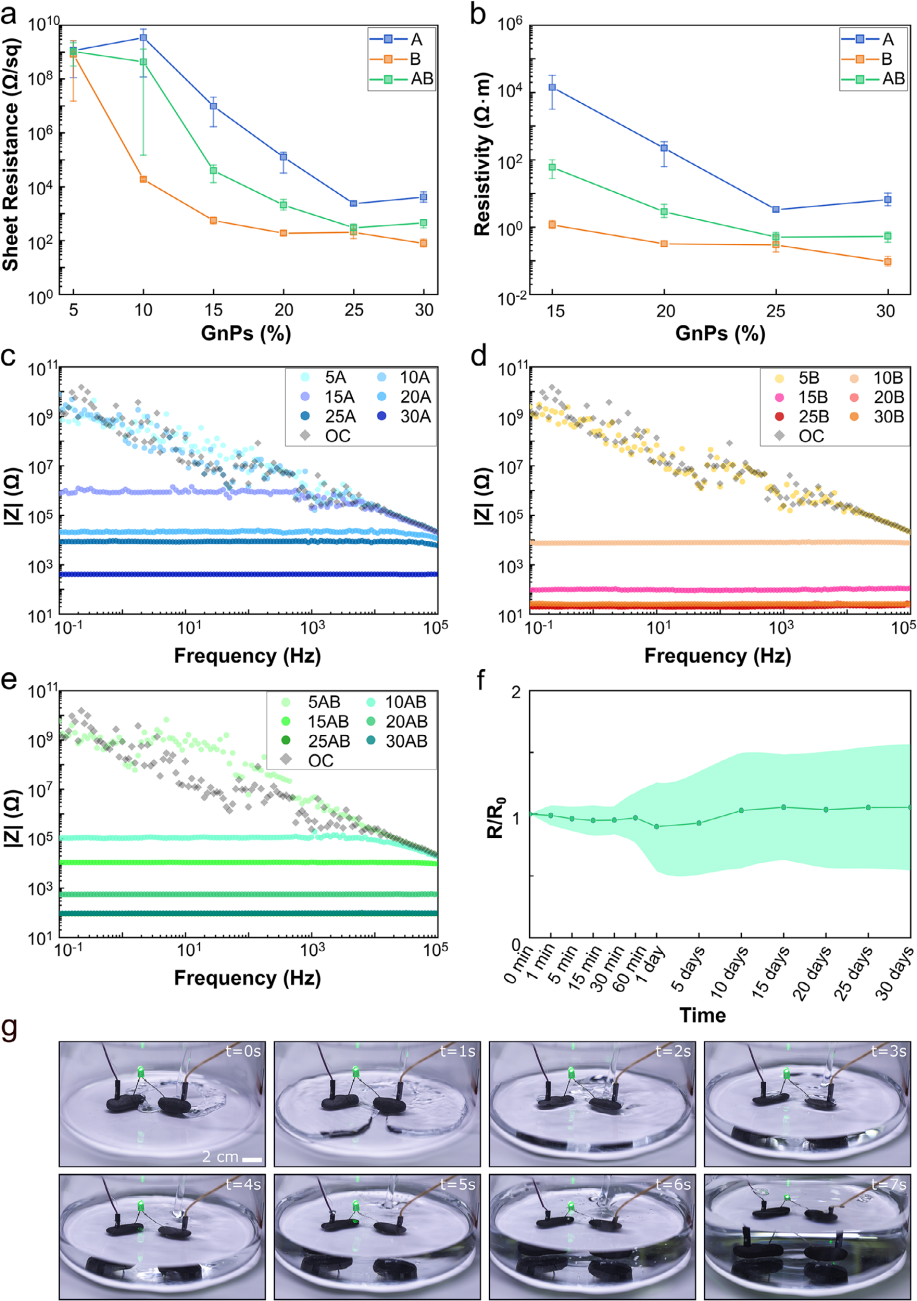
a,b) Sheet resistance and resistivity as a function of GnPs loadings for the two components before mixing (A with the initiator and B with the accelerator) and after crosslinking (AB paste). c,d,e) Impedance modulus for components A, B, and AB versus GnPs by wt.%, respectively. An open circuit (OC) measurement is reported as a reference. f) Resistance variation for pastes immersed in seawater for 30 days. g) Time‐lapse demonstrating the powering of an LED through the AB paste in the air and underwater.

The addition of GnPs was demonstrated to successfully impart electrical conductivity to the insulating matrix above a threshold loading value. The graph presented in **Figure** **2**a contains the sheet resistance data for samples of A, B, and AB with GnPs loads ranging from 5 to 30 wt.% and consistent thicknesses of ≈1.5 mm. The percolation threshold, marking the point at which the network formed by the GnPs becomes conductive ^[^ ^95^, ^96^, ^97^ ^]^ differs for the three materials. For component A, percolation occurs between 10 and 15 wt.%. By further increasing the filler loading, the sheet resistance of component A is reduced between 2000 to 4000 Ω sq^−1^ at 25 wt.%. Component B achieves percolation between 5 and 10 wt.%. and shows overall lower sheet resistance with respect to component A for a fixed filler loading, with a sheet resistance between 80 to 200 Ω sq^−1^ at 25 wt.%. Paste AB exhibits an intermediate behavior, with percolation reached between 10 and 15% wt.%. In the case of AB, the sheet resistance is reduced by increasing the filler concentration until a plateau is reached around 25 wt.%. of filler loading. Paste AB reaches a sheet resistance between 300 and 450 Ω sq^−1^, which lies in between those reported for components A and B separately.

The resistivities of the two components and the final paste, extracted by considering the sheet resistance data together with the thickness of the samples using Eq. (1), are displayed in Figure 2b. For formulations at 25 wt.%. GnPs loading, components A, B, and paste AB have a resistivity of 1.00, 0.01, and 0.10 Ω∙m, respectively. The resistivity values measured for the conductive paste align with the reported ranges for carbon‐based biopastes in the literature, typically spanning from 10^−4^ to 10^3^ Ω⋅m.^[^ ^95^, ^96^, ^97^, ^104^, ^112^, ^121^, ^122^ ^]^ Notably, these values also match those of conductive concretes incorporating carbon‐based fillers, which exhibit resistivity between 1 and 10^3^ Ω⋅m.^[^ ^50^, ^52^, ^53^, ^54^, ^56^, ^57^, ^58^, ^59^, ^60^, ^62^, ^63^, ^64^ ^]^

To gain a more comprehensive understanding of the electrical properties of the material, the bicomponent paste was analyzed in an alternating current regime. The electrical impedance moduli (|Z|) in the frequency range 10^−1^–10^5^ Hz for A, B, and AB at varied GnPs percentages are presented in Figure 2c–e, respectively, with the phases reported in Figure S3 (Supporting Information). The obtained percolation thresholds in DC are confirmed by these impedance measurements. The pastes have a purely resistive behavior after reaching the percolation threshold, showing the characteristic constant impedance modulus and phase magnitude of zero. Before percolation, the pastes behave as insulators, displaying a curve similar to that of an open circuit. With identical sample geometry across all pastes and the same electrical connection, at the highest GnPs loadings, component A exhibited an impedance modulus of ≈400 Ω, significantly higher than AB, which measured ≈90 Ω, while B demonstrated the lowest impedance modulus of ≈20 Ω. Furthermore, the electrical properties of AB were again observed to reach a plateau around 25 wt.%. GnPs concentrations, and the malleability of component A significantly declined with increasing nanofiller loadings. Thus, it was decided to test pastes with a 25 wt.%. GnPs content for further characterization since this nanofiller loading provided the best compromise in terms of electrical properties and processability.

Given the expected application requiring extended use in seawater, it is crucial to ensure that the paste can provide durable coral attachment underwater while maintaining conductivity and resisting water permeation. To evaluate this, the variation in electrical resistance of the crosslinked paste AB during 30 days of immersion in seawater was measured (Figure 2f), and how the impedance of the pastes was modified upon water immersion was assessed (see Figures S7 and S8, Supporting Information). The resistance variation was negligible throughout the period of observation, and the electrical impedance before and during water immersion showed no significant variations for component B and paste AB. In contrast, component A exhibited inferior performance, likely due to its more brittle texture and reduced cohesion, permitting water to penetrate the internal structure of the paste due to crack formation. Despite this, the outcomes prove excellent underwater stability of the electrical properties of the final, crosslinked AB paste. Figure 2g presents a time‐lapse of an LED being powered by a current that is passing through the AB paste. The LED remains illuminated both when the conductive paste is exposed to air and when it is submerged in tap water.

The pastes demonstrated hydrophobic behavior with GnPs loading ≥20 wt.% (Figure S5, Supporting Information). This figure presents water contact angle measurements for paste AB with GnPs loadings ranging from 15 to 30 wt.%. The hydrophobicity of the material is a property that could significantly contribute to its excellent water insulation.^[^ ^123^ ^]^ No significant macroscopic release of GnPs from AB paste into seawater was detected by analyzing the UV–vis spectra of the media after immersion of the bicomponent over 8 weeks, as shown in Figure S6 (Supporting Information).

Ensuring an appropriate hardening time for the bicomponent paste is crucial for corals' successful and durable attachment in an underwater environment. The hardening process must not be too rapid; allowing the operator sufficient time to shape the paste, position it correctly, and attach the selected coral fragment. Conversely, the hardening process should not be excessively slow, as the paste needs to withstand underwater currents and provide a firm substrate to support coral growth.

**Figure 3:**
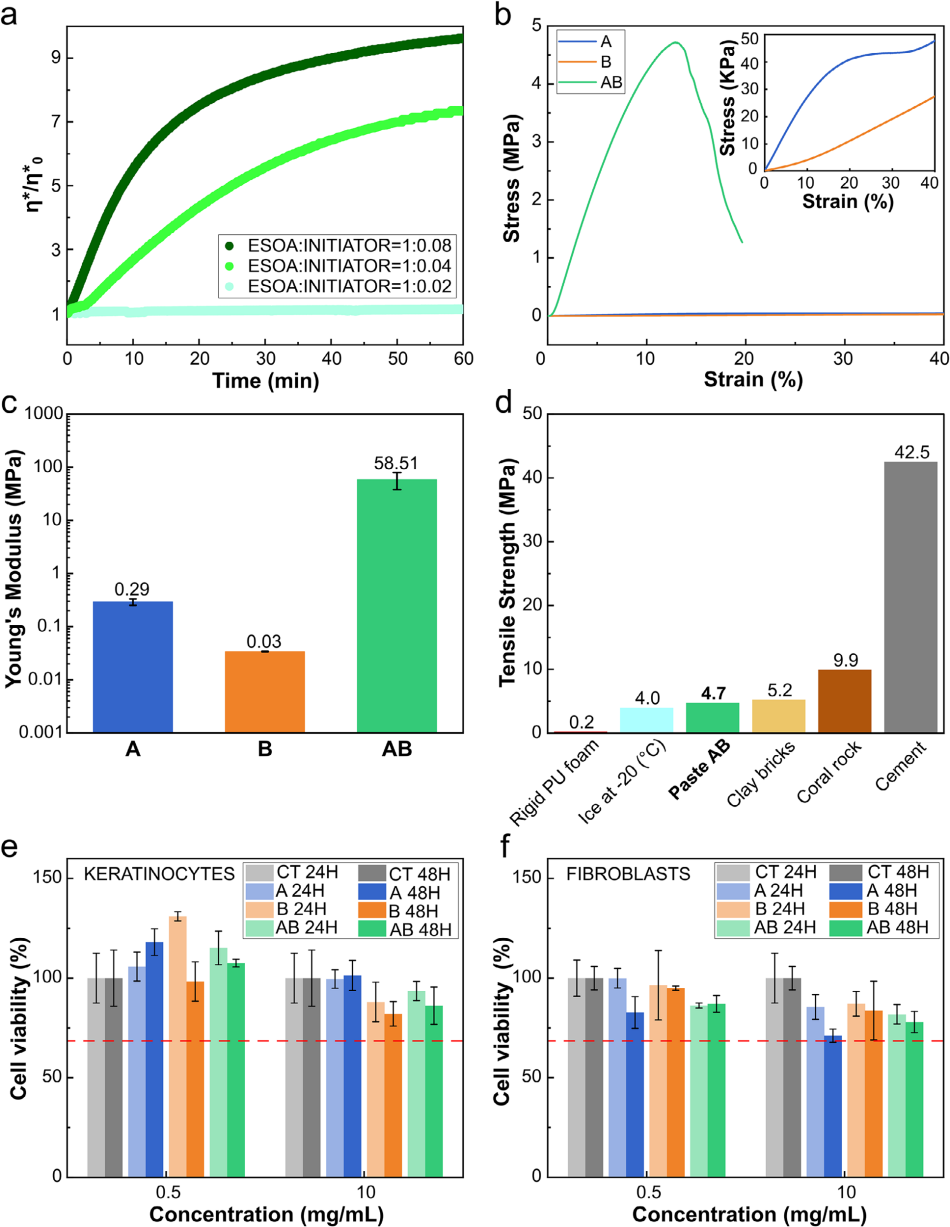
a) Complex viscosity variation during time sweep test. Different curves show results as a function of different initiator loadings. Pastes were tested during cross‐linking. b) Compressive tests’ mean curves for the two components and for the crosslinked paste. c) Mean compressive Young's moduli. d) Tensile strength comparison between different materials. e,f) Biocompatibility test on keratinocytes e) and fibroblasts f) for concentrations of 0.5 and 10 mg mL^−1^ at 24 and 48 h, respectively. CT = control.

Hence, to understand the viscoelastic properties of the pastes and the dynamics of the crosslinking reaction, rheological measurements were conducted. Measurements were performed with a time sweep test, keeping amplitude and frequency constant, as reported in **Figure** **3**a. Before that, amplitude and frequency sweep tests were conducted (Figure S9, Supporting Information) to extract the parameters required for the time sweep test. Further details are provided in the methods section. The crosslinking dynamics of paste AB, post‐mixing, were assessed employing three distinct ESOA:INITIATOR (weight:weight) quantities of 1:0.08, 1:0.04, and 1:0.02 in component A. The dark green curve represents the AB paste with the highest amount of initiator (1:0.08) and displays a considerably swifter progressive increase in the complex viscosity than the bright green curve, which represents the AB paste with an intermediate initiator amount (1:0.04). This difference signifies a more rapid crosslinking process for the higher initiator concentration. In contrast, the paste with the smallest initiator amount (1:0.02) shows a constant viscosity after mixing, which suggests the absence of crosslinking. These results imply the possibility of modulating the crosslinking dynamics of the mixed AB pastes by adjusting the initiator quantity. This adaptability is valuable for ensuring long‐term coral attachment for various coral types and under different environmental conditions. Indeed, settings with strong underwater currents and steep reef slopes would benefit from a fast hardening time that would enable the mixture to secure the coral fragments quickly. In contrast, transplanting larger coral colonies into flatter areas with minimal currents may require a slower hardening process to ensure marine biologists get sufficient time to position and secure the corals before the mixture sets.

Figure 3b–d presents the outcomes of mechanical characterization of the pastes via compression tests. In Figure 3b, the mean curves of the compression test for the different pastes are displayed, while the stress–strain curves for all the compression tests are presented in Figure S10 (Supporting Information). The results indicate that component B exhibits a softer consistency than component A (Figure 3c), with Young's moduli of 0.034 and 0.29 MPa, respectively. These results are in agreement with the qualitative observations on the consistency of the components made in Figure 1. Paste AB exhibits significantly greater stiffness compared to its components due to the crosslinking process within the ESOA matrix, imparting enhanced mechanical strength, rigidity, and durability. In particular, the Young's modulus of paste AB is equal to 58.51 MPa, a 2000‐fold increase for component B and a 200‐fold increase for component A (Figure 3c). Figure 3d reports the compressive strength of the paste AB after crosslinking, which is equal to 4.71 MPa. The hardened paste exhibits compressive strength comparable to that of clay bricks^[^ ^124^ ^]^ and falls within the same order of magnitude as coral rock,^[^ ^124^, ^125^, ^126^, ^127^, ^128^ ^]^ highlighting that the mechanical properties of the AB pastes obtained align well with the properties required for a substrate to facilitate successful coral attachment and growth.

To investigate the fate of the conductive biopaste in seawater, biochemical oxygen demand (BOD) measurements were conducted. Observation of microorganism activity in the seawater during the experiment (Figure S11, Supporting Information) confirmed that the vegetable oil‐based matrix of the paste is biodegradable. However, increasing GnPs content within the paste reduced its biodegradability. This trend aligns with previously reported findings in the literature.^[^ ^95^, ^97^, ^129^ ^]^ Graphene and its derivatives are generally considered chemically inert in many natural environments, though partial degradation can occur under certain biological and chemical conditions.^[^ ^120^ ^]^ Enzymatic processes have been shown to degrade graphene oxide,^[^ ^130^ ^]^ with the potential to enhance this degradability further through functionalization.^[^ ^131^, ^132^ ^]^ Some graphene nanomaterials undergo microbial degradation by specific fungi‐released enzymes^[^ ^133^ ^]^ and by environmental microbes in general.^[^ ^134^ ^]^ Furthermore, although the paste is not entirely biodegradable due to the presence of these carbon nanofillers, it's partial biodegradability is already a marked improvement over conventional hardening pastes that are known to be completely non‐biodegradable in seawater.^[^ ^114^ ^]^

Biocompatibility is a crucial characteristic for a paste that must be manually shaped and mixed by marine biologists or other human operators prior to application on corals. Thus, cytotoxicity tests for the conductive paste on keratinocytes (Figure 3e), and fibroblasts (Figure 3f) were performed. Cytotoxicity assays indicated the materials had no adverse effects on cell viability when exposed to human epithelial cells. Cell viability was higher than the 70% threshold after both 24 and 48 h in every concentration considered. Biocompatibility results for a wider range of paste concentrations are reported in Figure S12 (Supporting Information). Pictures of fibroblasts and keratinocytes during testing are displayed in Figure S13 (Supporting Information). From the morphological analysis of the cells, no alterations in the cell shape were noticed. Furthermore, the survival and growth of various coral species when attached to different substrates using the conductive biopaste were qualitatively evaluated. The bicomponent exhibited optimal biocompatibility with corals. Corals successfully grew and thrived while attached to the underwater paste, highlighting its safety and suitability for the intended application (Figure S14, Supporting Information).

**Figure 4:**
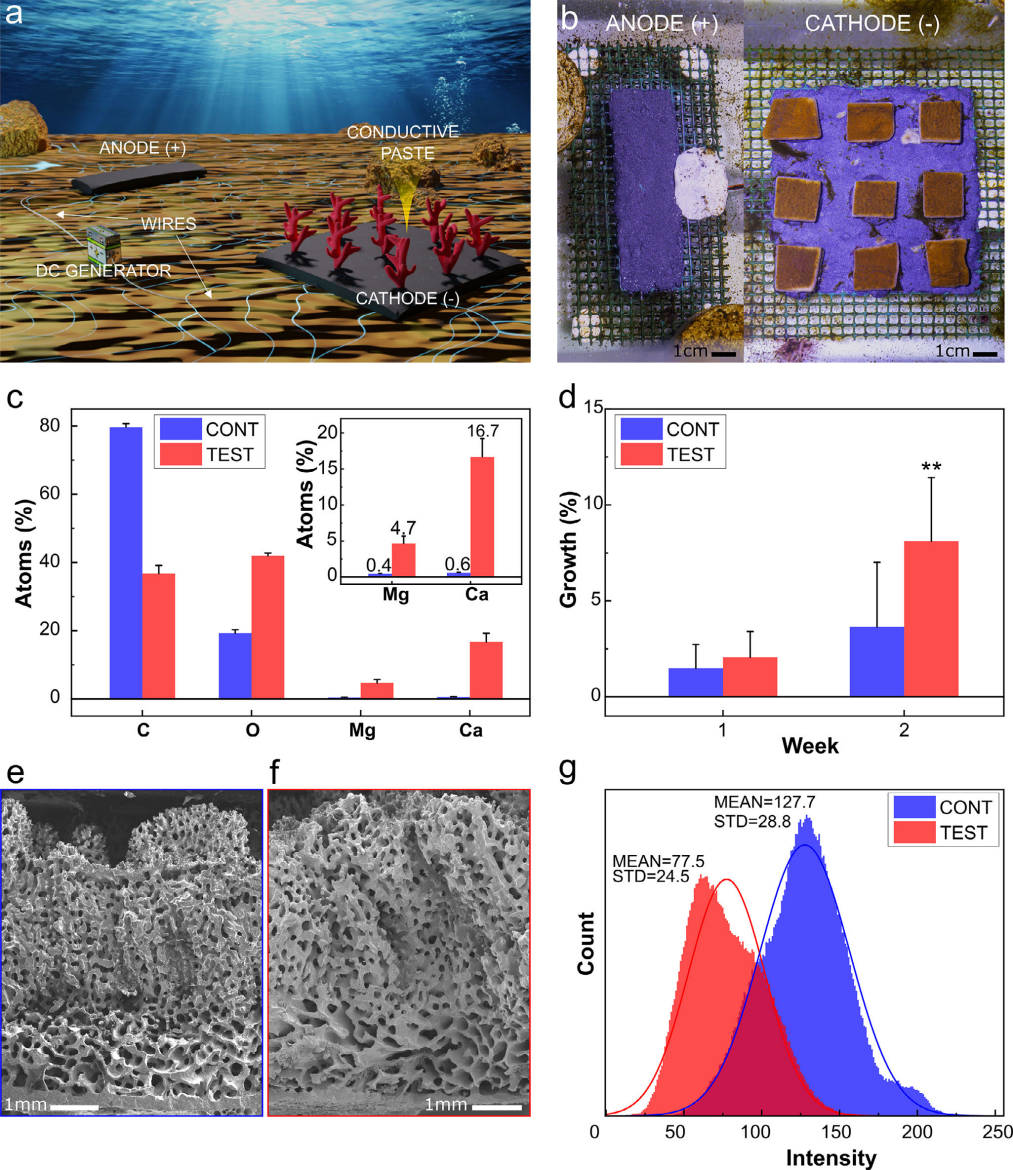
a) Scheme showcasing mineral accretion technology (MAT) setup. b) Anode and cathode made with the crosslinked AB paste for the MAT experiments in the aquarium. c) SEM‐EDS quantitative analysis results for the surface of the control mesh (CONT) versus surface of the cathode (TEST) after 8 weeks of immersion in water. Atom percentage is plotted for carbon, oxygen, magnesium, and calcium. d) Weekly percentage of growth for CONTROL and TEST for the 1st and 2nd weeks. Data are presented as mean ± SD (*n* = 15 for CONTROL and *n* = 15 for TEST; Student's *t*‐test ^*^: *p* < 0.05; ^**^: *p* < 0.01 and ^***^: *p* < 0.001). e,f) Cross‐sections at SEM of one representative control e) and test f) fragment after eight weeks of the experiment. g) Pixel intensity for control and test coral fragments.

Once the AB paste was confirmed to be biocompatible, MAT experiments^[^ ^39^, ^40^, ^41^, ^42^, ^43^, ^44^ ^]^ were conducted on corals at the Genoa Aquarium. A schematic representation as well as a photograph of the setup used are shown in **Figure** **4**a,b, respectively. Both the anode and the cathode were composed of the developed AB paste. Corals were then attached to the cathode using an additional amount of conductive biopaste. An AC‐DC converter supplied current to the anode and cathode via wires in contact with the electrodes but insulated from the surrounding seawater. When a potential difference is applied across the two electrodes, oxidation processes occur at the anode while reduction occurs at the cathode. At the anode, chlorine is generated, and a localized acidic environment is formed.^[^ ^40^ ^]^ To mitigate the potentially harmful effects of this acidic microenvironment on corals’ growth, the electrodes were placed on opposite sides of the tank, minimizing the influence of anode‐induced conditions on the corals. Control corals were attached in the same fashion as the test condition, but were positioned in another tank where no voltage was applied.

Preliminary experiments were conducted in order to identify the optimal MAT configuration as shown in Figures S15–S17 (Supporting Information). Following these experiments, and in agreement with previous MAT studies,^[^ ^43^, ^135^, ^136^ ^]^ current (*I*) was selected as the independent variable for further investigations. Specifically, a current of 5 mA, corresponding to the perception threshold for humans in DC, was selected.^[^ ^137^ ^]^ Notably, the current of 5 mA applied in this setup is much smaller compared to typical values of a few amperes reported in the literature. The low current was mainly due to the electrode's close spatial distribution and the AB paste's higher resistivity compared to metals, allowing for a greater potential difference (*ΔV*) to be maintained between the electrodes with a lower current. This results in a power consumption between 15 and 30 mW; values considerably lower than the 35–144 W typically reported in literature.^[^ ^41^, ^43^, ^44^, ^136^ ^]^ Given the cathode size, the current density was 0.5 A m^−2^, which is in line with values from previous studies (ranging from 0.01 to 30 A m^−2^).^[^ ^41^, ^43^, ^135^, ^136^ ^]^ However, a major requirement for the system was that the selected current for a MAT experiment must enable water electrolysis^[^ ^136^ ^]^ by sustaining a *ΔV* greater than 1.229 V. As shown in Figures S15 and S16 (Supporting Information), it was possible to reach and maintain functional values with the AB paste‐based system. Notably, this tension is still orders of magnitude lower than lethal levels reported in the literature for fish.^[^ ^138^ ^]^

Next, it was important to evaluate if electrochemical mineral accretion at the cathode, along with a zone of high calcium concentration surrounding it, was induced by the applied ΔV. To assess this, SEM energy dispersive X‐ray spectroscopy (EDS) analysis was conducted for the paste surfaces of both cathode and control meshes. As shown in Figure 4c, after eight weeks, the cathodes’ surfaces (TEST) exhibited substantial mineral accumulation, with calcium and magnesium accounting for 16.7% and 4.7% of atomic composition, respectively. In contrast, the control meshes (CONT) showed minimal deposition of these elements, with calcium and magnesium levels at just 0.6% and 0.4%, respectively. The accreted minerals on the cathodes were found to be magnesium hydroxide Mg(OH)_2,_ and calcium carbonate CaCO_3_. These outcomes align with what was reported in the literature^[^ ^40^ ^]^ however, the amount of accreted calcium on the cathode in this study exceeded the levels reported in a previous study,^[^ ^139^ ^]^ where calcium was observed ≈0.5% and magnesium at ≈25% after 12 weeks of MAT.

*Montipora foliosa* was selected as the coral species for MAT testing due to its common use as a model species for growth experiments in aquaria.^[^ ^140^, ^141^ ^]^ Figure S19 (Supporting Information) provides macro‐scale images of the coral fragments taken during the experiments, which lasted eight weeks. The percent area growth in the x‐y plane was evaluated every week. This parameter was selected as the variable to measure coral growth as it can be assessed directly from photographs taken during the experiment, thus minimizing stress on the coral specimens. Additionally, previous studies have validated area growth percentage as a reliable indicator of overall 3D growth in *Montipora* species.^[^ ^141^ ^]^

Significant differences in the results for the area of growth of the coral fragments were observed in the 2nd week (Figure 4d), with control fragments growing by 3.64% ± 3.37% and test fragments exposed to the electric field growing by 8.11% ± 3.31%, roughly a two‐fold increase. A *t*‐test confirmed that the growth difference between the test and control groups after two weeks was statistically significant (*p* = 0.0035). No additional significant differences were observed during the remaining six weeks of the experiment (Figure S20, Supporting Information). The accelerated early growth may be attributed to increased calcium availability at the cathode, which likely enhances the healing and growth of coral tissue, particularly during the initial stages of the experiment. Huang et al. have also reported observing a short‐term enhancement of the coral calcification rate when exposed to low electric current density; however, the effects diminished with prolonged exposure, showing no significant impact over an extended treatment duration.^[^ ^135^ ^]^

SEM cross‐section images of two coral fragments after the MAT experiments were analyzed to determine whether prolonged exposure to current within the MAT experiment causes changes in the basic mineral structure of corals. The results are presented in Figure 4e–f, which display the control coral fragment and the test fragment, respectively. Additional images can be found in Figure S21 (Supporting Information). These images reveal that the pore morphology and arrangement in the coral tissue under test and control conditions are similar, confirming no evidence of abnormal growth being induced by MAT.

Corals’ coloration is associated with their concentration of zooxanthellae, the symbiotic algae living inside their mineral structure that provide them with their pigmentation as well as energy through photosynthesis. Such coloration is considered an indicator of their health status.^[^ ^142^ ^]^ Specifically, a darker coloration typically reflects a higher density of zooxanthellae, whereas a lighter or more bleached appearance indicates a reduced concentration of these symbionts. To quantify the coloration of the tested corals, the pixel intensity^[^ ^143^ ^]^ of the photographs acquired for corals in the aquarium tanks for all test and control coral fragments was analyzed, where pixel values ranged from 0 (black) to 255 (white). The photographs were obtained with identical illumination conditions. Qualitatively, it was observed that the test coral fragments exhibited a darker and more vibrant coloration than the controls. This observation aligns with the quantitative analysis presented in Figure 4g, where the average pixel intensity of control fragments was 127.7 compared to 77.5 for test fragments. This indicates that the control fragments exhibit a lighter (whiter) coloration relative to the test fragments. One of the claims of other studies utilizing MAT technology is that it increases ATP concentration at the cathode, which enhances coral tissue health.^[^ ^39^ ^]^ This may explain the improved appearance and condition of the test corals observed in the experiment.

A crucial point in long‐term use of traditional MAT is mineral buildup on the cathode, which can eventually insulate the electrode, blocking the redox reaction that yields electrolysis,^[^ ^44^ ^]^ but also protecting against corrosion. This creates a trade‐off between performance and durability. The biopaste has the possibility to allow controlled, on‐demand MAT application without having to rely on accreted minerals for protection; thereby avoiding the risk of cathode insulation over time. As previously discussed, in a MAT setup to maintain the necessary *ΔV* to induce seawater electrolysis, materials with higher conductivity, such as metals, require higher currents that can have negative effects on corals. This issue becomes more pronounced in small setups, such as the one presented in this work, where the electrical resistance of the electrodes is reduced due to their small size. Therefore, the electrical properties of the electrodes are another important factor in MAT. An advantage of the presented AB biopaste is its tunable resistance, adjusted by varying the conductive filler content, that offers an additional pathway for optimizing MAT performance to suit unique application conditions. Moreover, it offers an effective approach for miniaturizing MAT in an *ex situ* setup by taking advantage of its easy positioning and moderately low conductivity, ultimately enhancing coral fragments' growth and accelerating coral restoration efforts. Additional images of *Montipora* fragments anchored on the bicomponent substrate are provided in Figure S22 (Supporting Information). These images were taken one year after initial positioning and more than six months after the conclusion of the MAT experiment. The corals continued to grow and colonize the substrate, with fragments from the same colony eventually fusing. This outcome demonstrates the potential of this technology to support long‐term coral growth even without a continuous power supply.

**Table** **1** presents a comparison of key characteristics of relevant materials from the literature with the conductive biopaste presented in this study. The developed AB biopaste is observed to uniquely combine outstanding properties: it hardens effectively, supports coral attachment on various substrates, such as coralline rock, mortars, concretes, and plastics, exhibits electrical conductivity, does not release toxic compounds, and is not subjected to rust formation underwater. This combination of properties highlights that the presented system is outperforming all other solutions reported in the literature. The AB biopaste provides a versatile and eco‐friendly solution for coral restoration that can potentially be employed in combination with other effective coral restoration techniques, such as microfragmentation, a method used to farm slow‐growing corals after fragmenting coral colonies in smaller pieces, often starting with an *ex situ* phase. Microfragmentation has been adopted in projects aimed at cultivating many fragments per year. The integration of microfragmentation, coral attachment, and MAT combined with the possibility of direct application of the AB paste on reefs, as illustrated in Figure S23 (Supporting Information), could offer significant potential to scale up coral restoration efforts extensively. The development and application of eco‐friendly, advanced materials, such as the novel conductive hardening paste presented in this study, thus offers a transformative potential for addressing critical challenges facing coral reefs. By mitigating issues like coral diseases and coral wounds,^[^ ^144^ ^]^ supporting recovery from bleaching events,^[^ ^145^ ^]^ and enhancing restoration efforts,^[^ ^114^, ^146^ ^]^ these materials provide a more effective way to protect and preserve these vital ecosystems.

**Table 1:** Comparison of key characteristics of relevant materials for coral attachment and mineral accretion technology.

Materials	Resistivity [Ω⋅m]	Biodegradability	Release of toxic compounds	Compression Strength [MPa]	Employable for coral attachment	Adjustable crosslinking
Epoxy^[^ ^114^ ^]^ bicomponents	Not conductive	No	Yes	15	Yes	No
Marine^[^ ^147^ ^]^ Concretes	Not conductive	No	No	30	Yes	No
Coralline^[^ ^127^ ^]^ rock	Not conductive	No	No	10	No	No
Metals^[^ ^148^, ^149^, ^150^, ^151^ ^]^ (steel/iron)	10^−7^ to 10^−6^	No	Yes	200–1000	No	No
Metal^[^ ^83^, ^84^, ^85^, ^87^, ^88^, ^89^, ^90^, ^91^ ^]^ biopastes	10^−5^ to 10^−2^	Yes	No	<1	No	No
Carbon^[^ ^102^, ^103^, ^107^, ^109^, ^110^, ^111^, ^112^ ^]^ biopastes	10^−4^ to 10^3^	Yes	No	<1	No	No
Conductive underwater biopaste	10^−1^	Yes	No	5	Yes	Yes

## 3 Conclusion

In pursuit of facilitating improvements in coral reef restoration strategies, a bicomponent conductive paste designed for application in underwater environments was developed. In particular, the paste was used for coral attachment and electrochemically driven mineral accretion technology (MAT). The paste was realized with a bio‐based and biodegradable matrix of soybean oil epoxidized acrylate and graphene nanoplatelets, commercially available and cheap carbon‐based conductive fillers. Upon mixing the two components, one containing an initiator and one containing an accelerator, the paste underwent crosslinking. When immersed in seawater, the paste showed stable electrical properties essential for marine applications. Furthermore, compression tests confirmed that the crosslinked paste has superior rigidity and compressive strength, comparable to coral rock, an essential characteristic for supporting coral adhesion and growth. It was additionally demonstrated that the paste's crosslinking dynamics could be modified by controlling the initiator concentration.

The paste demonstrated non‐cytotoxic behavior on human epithelial cells and high biocompatibility with corals. MAT experiments conducted on live corals using the conductive biopaste over eight weeks indicated that the paste facilitates electrochemical mineral accretion and enhances coral growth in early stages, with no observed structural changes in coral morphology. The results showed a significant difference in growth between test coral fragments and controls by the 2nd week, with test fragments subjected to the MAT showing an 8.11% increase in growth compared to a 3.64% increase in the control fragments. Additionally, coral fragments exposed to electric fields showed enhanced color intensity that potentially indicates improved health. Evaluation of key properties of materials presented in the literature compared to those of the developed conductive biopaste demonstrated its enhanced effectiveness for coral restoration.

This work shows how the interdisciplinary combination of engineering, materials, and marine sciences can help to address critical challenges facing coral reef preservation and restoration. In future work, more extensive MAT experiments involving a broader range of coral species with different growth morphologies and rates, extended observation periods, and different current densities on the cathode could be conducted. Additionally, it may be beneficial to focus on evaluating the feasibility of implementing the technology in artificial and real reefs. Further consideration can also be given to evaluating its performance in combination with microfragmentation to further accelerate coral fragment growth. This will allow for a more comprehensive assessment of the presented technology's long‐term performance, scalability, and practicality; building upon the promising results obtained in the present study.

As a final consideration, a reliable, renewable, in situ energy harvesting solution will be essential to deploy this technology across vast reef areas. Modular solar panels or submersible turbines could be installed and removed as needed, providing power during crucial moments like active attachment, initial growth phases, and bleaching events, given the potential effectiveness of MAT in combating coral stress. Integrating these adaptive, renewable energy systems will be critical for implementing the developed technology on‐site.

## 4 Experimental Section

### Materials

Epoxidized soybean oil acrylate (ESOA) and lauroyl peroxide were purchased from Sigma–Aldrich. Para‐toluidine ethoxylate tertiary amine, known commercially as Bisomer PTE, was supplied by GEO Specialty Chemicals. Graphene nanoplatelets (6–8 nm thick × 25 µm wide) were bought from Strem Chemicals.

### Conductive Biopaste Preparation

The conductive biopaste is formulated as a bicomponent system (A + B). Component A includes the radical initiator lauroyl peroxide, while component B contains the accelerator para‐toluidine ethoxylate tertiary amine. Components A and B were mixed using a THINKY Mixer (https://www.thinkymixer.com/en‐us/). The mixing process involved a program set to mix at increasing speed for 90 s in a plastic cup provided by THINKY.

Initially, a small quantity of the radical initiator was mixed with ESOA to form component A using weight‐to‐weight ratios ranging from 1:0.02 to 1:0.08 (ESOA: INITIATOR). For component B, the accelerator was combined with ESOA in a 1:0.08 w/w.

For component A, the radical initiator was pre‐dissolved in ESOA by heating in an oven at ≈50°C for 15–30 min before mixing. As demonstrated in Figure S24 (Supporting Information), the thermogravimetric analysis of the crosslinked paste indicates degradation onset around 350 °C, confirming that the heating step did not affect the ESOA properties.

GnPs were then incorporated as fillers into components A and B to impart electrical conductivity and increase viscosity, yielding a paste‐like consistency in the final formulations. GnPs were used in amounts ranging from 5% to 30% of the total biopaste weight. The hardened conductive biopaste was formed by hand‐mixing components A and B in equal amounts for ≈1 min, followed by a 20–40 min curing period.

### Methods

Unless otherwise specified, at least three measurements on different samples were performed for each technique described below.

### Scanning Electron Microscopy

The sample surface morphology and cross‐section were examined using a scanning electron microscope (SEM). Specifically, a variable pressure JEOL JSM‐6490LA (JEOL, Tokyo, Japan) SEM with a tungsten thermionic electron source, operating in high vacuum at an acceleration voltage equal to 5 kV was used. Before imaging, samples were coated with a 10 nm gold layer employing a Cressington 208 HR Sputter Coater (Cressington, Watford, UK). Cross sections were obtained by immersing the samples in liquid nitrogen and breaking them in half.

### Attenuated Total Reflection‐Fourier Transform Infrared (ATR‐FTIR) Spectroscopy

Infrared spectra were collected using an ATR diamond crystal accessory (MIRacle ATR, PIKE Technologies) connected to an FTIR spectrometer (Vertex 70v FT‐IR Bruker). The spectra were collected over the range of 4000–600 cm^−1^. The resolution was 4 cm^−1^, and 128 scans were accumulated for each measurement.

### Percolation Study and Resistivity Computation

Fixed amounts of conductive pastes A, B, and AB were manually spread on glass slides at a uniform and constant thickness. Silver ink (‘Silver conductive paint 186–3600’ by RS Pro) contacts were applied to each sample, with four parallel lines drawn on each one.

*I*–*V* curves were measured using a Keithley 2612A sourcemeter, and resistances (*R*) were determined through linear fitting of the curves. This analysis was conducted at increasing GnPs loading to investigate the percolation threshold of the various pastes.

SEM images were captured for the cross‐section of each sample to measure their thickness, which was equal to 1.5 ± 0.5 mm for all samples.

The resistivity (*ρ*) was calculated using the geometrical data obtained from SEM images, applying the following formula:

(1) $$\rho =R\frac{wt}{L}$$

where *R* is the resistance, *w* is the width, *t* is the thickness, and *L* is the distance between the probe tips. The contacts were drawn such that *w* was equal to *L*.

### Resistance Stability Underwater

To study if the electrical properties of the hardened paste were stable upon prolonged immersion in seawater, a stability analysis was performed. Fixed amounts of components A and B were mixed, wires were immersed in the paste before crosslinking, and, after crosslinking, the pastes were positioned in containers filled with seawater. Measurements of the resistance for four different pastes were taken at regular intervals for 30 days. A picture of the employed setup is shown in Figure S4 (Supporting Information).

### Impedance ‐AC Measurement

For impedance measurements, samples were prepared on glass slides as described above, and electrical contacts were applied using the same silver ink. The distance between two adjacent electrodes was 5 mm, and the width of each electrode was also 5 mm. The impedance was measured using a potentiostat (Multi‐PalmSens 4) by applying a sinusoidal stimulus of 200 mV within a frequency range of 0.1 Hz–100 kHz. Impedance measurements in air were obtained by contacting the sample with two micro‐positioning probes (EB‐700 by Everbeing Int'l Corp). Underwater impedance measurements were obtained by attaching the sample to the bottom of a large petri dish (200 × 30 mm^2^) using Kapton tape. The sample was then contacted using the same micro‐positioning probes, and the petri dish was filled with water (MilliQ millipore ultrapure water, tap water, and seawater collected from Genoa Aquarium, Italy). It is worth noticing that in this specific case, electrical contacts are exposed to water, potentially creating an additional parallel path. To reduce this effect, an alternative setup where electrical wires were encapsulated in the biopaste was also investigated. For this setup, fixed amounts of components A and B were manually mixed to form spherical samples. Two standard copper wires were immersed in the paste before completion of crosslinking so that the connection point between the sample and the electrical wire remained effectively encapsulated after crosslinking. Samples were then positioned in containers filled with water and connected to the potentiostat using crocodile clips affixed directly onto the other end of the encapsulated wires. In this configuration, the connection between the sample and the wire was not directly exposed to water.

### Water Contact Angle

Water contact angles (WCA) of the conductive biopaste were determined with a contact angle goniometer OCA‐20 (DataPhysics Instruments GmbH, Filderstadt, Germany) operating at room temperature. Droplets of deionized water, each 5 µL in volume, were carefully placed on the surface, and the contact angle was determined using specialized software. To ensure reliability, seven separate measurements were conducted for each sample.

### Stability, Underwater Release with UV–VIS

The cured biopaste, designated as AB, was carefully transferred into 10 mL Falcon tubes and submerged in seawater before sterilization under UV light to inhibit algae and bacteria growth. Control vials containing only seawater were concurrently prepared. To prevent algae and bacterial proliferation, all samples were securely sealed and refrigerated until the day of measurement. Measurements were scheduled at intervals of 0, 2, 4, and 8 weeks from the test's initiation. For each measurement, a pair of vials—one control and one test—were utilized. Seawater samples from each Falcon were withdrawn and analyzed employing a Varian Cary 6000i Scan UV–vis spectrophotometer (Walnut Creek, California, USA). Transparency assessments were conducted by measuring the absorbance at 600 nm, in particular, the transparency of the solutions was determined using the formula: 𝑇(%) = 10 − 𝐴𝑏𝑠 ∗ 100.

### Compression Test

The mechanical properties of components A, B, and the AB paste were measured through uniaxial compression employing a 3365 Instron dynamometer. The pastes were molded into small cylinders with an approximate height of 10 mm and a diameter of 10 mm, then subjected to testing at a displacement rate of 5 mm min^−1^. From the evaluation of the stress–strain curves the Young's modulus and the strength of the samples were extracted.

### Rheological Tests

Rheological measurements were conducted using an Anton Paar Modular Compact Rheometer (MCR 302). Initially, amplitude sweep tests were carried out on components A and B, with component A being tested under varying initiator loadings. These amplitudes sweep tests (Figure S9, Supporting Information), conducted at an angular frequency of 10 rad s^−1^, were essential for determining the Linear Viscoelastic Region, which is defined as the range in which the sample's structure remains intact under testing conditions. This determination was necessary for the execution of subsequent tests. The shear strain range employed for the amplitude sweep tests was between 0.001% and 10%. After establishing the Linear Viscoelastic Region, an amplitude of 0.05% was selected for the frequency sweep tests, which spanned from 0.1 to 100 rad s^−1^. Subsequently, to study the crosslinking dynamics of the paste, time sweep tests were performed for 60 min at a constant shear strain of 0.05% and an angular frequency of 10 rad s^−1^. Before crosslinking, components A and B were mixed for 2 min and positioned under the rheometer plates. The crosslinking dynamics of the paste were assessed with different initiator loadings by employing ESOA:INITIATOR ratios (weight:weight) of 1:0.02, 1:0.04, and 1:0.08 in component A.

### Biochemical Oxygen Demand Test

The biodegradability of the pastes was assessed using the biochemical oxygen demand (BOD). For each sample, around 200 mg of material was inserted into 432 mL of seawater collected from the Porto Antico area in Genoa, Italy. Collection of water from a natural environment allows for utilization of pre‐existing microbial populations and their supporting saline nutrients, ensuring the most realistic experimental conditions possible. To promote microbial decomposition, the experiment was carried out at room temperature in 510 mL amber glass bottles sealed with OxiTop measuring heads.

Sodium hydroxide tablets were used to capture the CO₂ produced during biodegradation. Biotic oxygen consumption within the system's free volume was determined from the observed pressure decrease. Triplicates were employed for each sample. After 30 days, raw oxygen consumption readings (mg O_2_/L) were adjusted by subtracting the average blank values, such as the oxygen uptake measured in seawater without any test material. The corrected oxygen consumption was then normalized to the sample mass and expressed per 100 mg of material (mg O₂/100 mg). Triplicate means were calculated and plotted against exposure time. The BOD was determined as the difference between the initial and final dissolved oxygen concentrations, reported in mg O₂/L of water (mg/L).

To visualize the data effectively, a scatter plot was created using every 12th point of the calculated BOD values. To emphasize underlying trends, smoothed BOD curves were also generated via a low‑pass filter.

### Thermal Analysis

The thermal degradation properties of the biopaste were examined through thermogravimetric analysis (TGA) employing a TGA Q500 instrument (TA Instruments, USA). The experiments utilized around 4 mg of sample within a platinum pan. Measurements were conducted under an inert nitrogen flow of 50 mL min^−1^ over a temperature range from 30 to 800 °C. A consistent heating rate of 10 °C min^−1^ was maintained. The weight decrease and its first derivative as a function of time and temperature variations were simultaneously recorded.

### Biocompatibility

Cytotoxicity assessment was conducted on HaCaT (keratinocytes) and HFF‐1 (fibroblast) cells. Biocompatibility on HaCaT was determined using the CellTiter‐Glo Luminescent viability assay (Promega Italia S.r.l., Milan, Italy). For each cell type, 96‐well plates were prepared in parallel. HaCaT and HFF‐1 cells were each seeded at 4  × 10^5^ cells/well in 100 µL of medium and incubated until the desired confluence was reached. After 24 h, cells were rinsed with warm PBS containing Ca^2+^ and Mg^2+^, and the medium was then replaced with one that contained the conductive biopaste at increasing concentrations (0.5, 1, 5, and 10 mg mL^−1^). Cells were incubated for a further 24 and 48 h. Control samples were handled with blank medium processed in the same manner as the biopaste‐containing samples. Cell viability was obtained by quantifying ATP levels by CellTiter‐Glo assay, and reported as the percentage of surviving cells relative to the control. Data is displayed as mean ± SD of three independent assays. Following ISO10993‐5 guidelines, since the cell viability of the samples was greater than 70% of the control group, all concentrations of conductive paste were considered biocompatible. The effect of the conductive paste on cell morphology was also checked with a LEICA DMI6000B inverted microscope.

### Mineral Accretion Experiments at Genova Aquarium

Mineral accretion experiments were conducted at the Genova Aquarium employing corals grown and reproduced inside the Aquarium's facilities. *Montipora Foliosa* was selected as the test species for all experiments, with 15 fragments used for tests, and 15 fragments from 6 donor colonies were used as controls. All fragments had an initial diameter of 1.25 ± 0.25 cm. The corals were cut into fragments of the appropriate size using a saw.

The experimental setup consisted of two 400 L glass tanks (one for the test and one for the control). The filtration system (composed of a sand filter, UV filter, and a protein skimmer) ensured a complete seawater change every 3 h. The illumination was set up at 13:11 h light:dark with an LED X lamp (240 W, 11000 K, 250 PAR) for both test and control samples. The same water was employed to fill both tanks, and the conditions of oxygenation and internal water circulation were identical. In both tanks, no additional nourishing substances were administered to the corals, such as *Artemia salina* or calcium.

The experimental corals were affixed using the conductive bicomponent paste to cathodes fabricated with the same paste, each measuring 10 × 10 cm. The corresponding anodes measured 10 × 3 cm and were made using the same conductive paste. The electrodes were positioned on opposite sides of the tank to minimize the effects of acidic conditions and chlorine production at the anode on coral growth. The anode was placed near the tank's outflow to further mitigate adverse conditions affecting the corals attached to the cathode. An AC‐DC converter supplied current to the electrodes via wires immersed in the conductive paste. A current of 5 mA was applied across the electrodes. Control corals were similarly attached with the bicomponent paste to a mesh coated with the AB paste matching the cathode dimensions, but no electricity was applied.

Corals were kept in the tanks for 56 days and their growth was measured every 7 days. The growth was measured with the software ImageJ, computing the areas of the corals in the x and y planes. In Figure S18 (Supporting Information), two examples are provided that illustrate the selection of coral fragment areas using ImageJ software.

### Color Intensity Evaluation

To quantify color intensity in coral fragments, pixel intensity values from images of both test and control fragments were analyzed. To achieve this, each pixel was assigned a grayscale value ranging from 0 (black) to 255 (white). The intensity distributions for all pixels were plotted on a single graph, followed by Gaussian interpolation of the test and control data. To ensure consistency, all coral fragments were briefly placed in the same tank under uniform lighting conditions before imaging.

### Statistical Analysis

Before statistical analysis of the MAT experiment, outliers were removed from both control and test populations by excluding the maximum and minimum values for each week. No data normalization or transformation was applied. Results are presented as mean ±SD for each time point. A two‐sided Student's *t*‐test was performed to compare the control group (*n* = 15) and the test group (*n* = 15), with the significance level set at *α* = 0.05. All statistical analyses were conducted using MATLAB R2023a.

## Conflict of Interest

The authors declare no conflict of interest.

## Supporting information



Supporting Information

## Data Availability

The data that support the findings of this study are available from the corresponding author upon reasonable request.

## References

[1] E. R. Rhodes , H. Naser , Natural Resources Management and Biological Sciences, IntechOpen Limited, United Kingdom, 2021.

[2] Great_Barrier_Reef_Foundation , Great Barrier Reef's Challenges and Solutions, https://www.barrierreef.org, (accessed: June 2025).

[3] NOAA_Office_For_Coastal_Management , Coral Reef Conservation Program, https://coast.noaa.gov, (accessed: June 2025).

[4] O. Hoegh‐Guldberg , L. Pendleton , A. Kaup , Reg. Stud. Mar. Sci. 2019, 30, 100699.

[5] O. Hoegh‐Guldberg , E. S. Poloczanska , W. Skirving , S. Dove , Front. Mar. Sci. 2017, 4, 252954.

[6] O. Hoegh‐Guldberg , in Coral Reefs: An Ecosystem in Transition, Springer, Berlin, 2010.

[7] UN_Environment_Program , A Path Toward a Resilient Planet, https://www.unep.org, (accessed: June 2025).

[8] E. Bayraktarov , P. J. Stewart‐Sinclair , S. Brisbane , L. Boström‐Einarsson , M. I. Saunders , C. E. Lovelock , H. P. Possingham , P. J. Mumby , K. A. Wilson , Restor. Ecol. 2019, 27, 981.

[9] L. Bostrom‐Einarsson , R. C. Babcock , E. Bayraktarov , D. Ceccarelli , N. Cook , S. C. A. Ferse , B. Hancock , P. Harrison , M. Hein , E. Shaver , A. Smith , D. Suggett , P. J. Stewart‐Sinclair , T. Vardi , I. M. McLeod , PLoS One 2020, 15, 0226631. 10.1371/journal.pone.0226631 PMC6992220 31999709

[10] M. Omori , Mar. Biol. Res. 2019, 15, 377.

[11] N. Epstein , R. P. M. Bak , B. Rinkevich , Restor. Ecol. 2001, 9, 432.

[12] G. Levy , L. Shaish , A. Haim , B. Rinkevich , Ecol. Eng. 2010, 36, 560.

[13] B. Rinkevich , Restor. Ecol. 1995, 3, 241.

[14] B. Rinkevich , Mar. Biol. 2000, 136, 807.

[15] L. Shaish , G. Levy , E. Gomez , B. Rinkevich , J. Exp. Mar. Biol. Ecol. 2008, 358, 86.

[16] R. Osinga , M. Schutter , B. Griffioen , R. H. Wijffels , J. A. Verreth , S. Shafir , S. Henard , M. Taruffi , C. Gili , S. Lavorano , Mar. Biotechnol. 2011, 13, 658. 10.1007/s10126-011-9382-7 PMC3159950 21584662

[17] P. Kružić , P. Sršen , L. Benković , Facies 2012, 58, 477.

[18] K. R. N. Anthony , D. I. Kline , G. Diaz‐Pulido , S. Dove , Proc. Natl. Acad. Sci. USA 2008, 105, 17442. 18988740 10.1073/pnas.0804478105 PMC2580748

[19] T. F. Cooper , J. P. Gilmour , K. E. Fabricius , Coral Reefs 2009, 28, 589.

[20] R. Izumi , E. S. Tan , H. Higa , Z. Shi , Y. Takeuchi , N. Isomura , A. Takemura , Coral Reefs 2023, 42, 385.

[21] T. Rothig , S. M. Trevathan‐Tackett , C. R. Voolstra , C. Ross , S. Chaffron , P. J. Durack , L. M. Warmuth , M. Sweet , Glob Chang Biol 2023, 29, 4731. 37435759 10.1111/gcb.16859

[22] C. Ferrier‐Pagès , J. P. Gattuso , Coral Reefs 2000, 19, 103.

[23] L. D. Smith , J. Exp. Mar. Biol. Ecol. 1998, 235, 147.

[24] S. C. A. Ferse , Restor. Ecol. 2010, 18, 399.

[25] C. A. Page , E. M. Muller , D. E. Vaughan , Ecol. Eng. 2018, 123, 86.

[26] L. Musco , F. Prada , G. D'Anna , N. M. Galasso , C. Pipitone , T. Vega Fernández , F. Badalamenti , Ecol. Eng. 2017, 98, 206.

[27] J. A. Hollarsmith , in 12th Int. Coral Reef Symp., Scripps Institution of Oceanography, San Diego, 2012.

[28] J. D. Unsworth , D. Hesley , M. D'Alessandro , D. Lirman , Restoration Ecology 2020, 29, 13299.

[29] E. Gomez , A. Edwards , R. Dizon , in Reef Rehabilitation Manual, (Ed: A. J. Edwards ), Coral Reef Targeted Research and Capacity Building for Management Program, Brisbane, Australia, 2010, 6.

[30] Y. Jiang , Chem. Soc. Rev. 2024, 53, 624.38109059

[31] J. A. Gonzalez , A. R. Histed , E. Nowak , D. Lange , S. E. Craig , C. G. Parker , A. Kaur , S. Bhuvanagiri , K. J. Kroll , C. J. Martyniuk , N. D. Denslow , C. S. Rosenfeld , J. S. Rhodes , Horm Behav 2021, 136, 105043. 34507054 10.1016/j.yhbeh.2021.105043

[32] X. Liu , H. Shi , B. Xie , D. D. Dionysiou , Y. Zhao , Environ. Sci. Technol. 2019, 53, 10188. 31393116 10.1021/acs.est.9b02834

[33] R. M. Dizon , A. J. Edwards , E. D. Gomez , Aquat. Conserv.: Mar. Freshw. Ecosyst. 2008, 18, 1140.

[34] P. Bilalis , A. Alrashoudi , H. H. Susapto , M. Moretti , S. Alshehri , S. Abdelrahman , A. Elsakran , C. A. E. Hauser , ACS Appl. Mater. Interfaces 2023, 15, 46710. 37768145 10.1021/acsami.3c10726

[35] E. Walton , L. Badder , C. T. Galindo‐Martínez , D. B. Berry , M. Tresguerres , D. Wangpraseurt , Front. Mar. Sci. 2024, 11, 1454887.

[36] H. M. Guzman , Bull. Mar. Sci. 1989, 44, 1186.

[37] W.‐C. Dullo , Facies 2005, 51, 33.

[38] K. P. Helmle , R. E. Dodge , P. K. Swart , D. K. Gledhill , C. M. Eakin , Nat. Commun. 2011, 2, 215. 21364554 10.1038/ncomms1222

[39] T. J. Goreau , Front. Mar. Sci. 2022, 9, 805113.

[40] W. Hilbertz , J. Ocean. Eng 1979, 4, 94.

[41] E. M. Borell , S. B. Romatzki , S. C. Ferse , Coral Reefs 2009, 29, 191.

[42] T. J. Goreau , Nat. Res. 2014, 05, 527.

[43] S. B. C. Romatzki , Mar. Biol. Res. 2014, 10, 449.

[44] M. G. Sabater , J. Exp. Mar. Biol. Ecol. 2004, 311, 355.

[45] M. Li , Y. Wang , Z. Liu , Y. Sha , G. V. Korshin , Y. Chen , Water Res. 2020, 175, 115675. 32155486 10.1016/j.watres.2020.115675

[46] P. Sarin , V. L. Snoeyink , J. Bebee , K. K. Jim , M. A. Beckett , W. M. Kriven , J. A. Clement , Water Res. 2004, 38, 1259. 14975659 10.1016/j.watres.2003.11.022

[47] S. Zhang , Y. Tian , Y. Guo , J. Shan , R. Liu , Chemosphere 2021, 262, 127904. 32799153 10.1016/j.chemosphere.2020.127904

[48] H. S. Lim , S.‐N. Kim , J. A. Lim , S.‐D. Park , J. Mater. Chem. 2012, 22, 20529.

[49] J.‐X. Wu , C.‐P. Chu , Y.‐C. Liao , J. Taiwan Inst. Chem. Eng. 2023, 142, 104616.

[50] F. Azhari , N. Banthia , Cem. Concr. Compos. 2012, 34, 866.

[51] S. Bai , L. Jiang , Y. Jiang , M. Jin , S. Jiang , D. Tao , Adv. Cem. Res. 2020, 32, 45.

[52] S. Bai , L. Jiang , N. Xu , M. Jin , S. Jiang , Constr. Build. Mater. 2018, 164, 433.

[53] V. Cerny , G. Yakovlev , R. Drochytka , S. Baranek , L. Meszarosova , J. Melichar , R. Hermann , Materials 2021, 14, 7505. 34947100 10.3390/ma14247505 PMC8705280

[54] B. Chen , K. Wu , W. Yao , Cem. Concr. Compos. 2004, 26, 291.

[55] F. Collins , J. Lambert , W. H. Duan , Cem. Concr. Compos. 2012, 34, 201.

[56] G. Goracci , S. D. J , Materials 2020, 13, 275. 31936238 10.3390/ma13020275 PMC7013725

[57] M. Krystek , A. Ciesielski , P. Samorì , Adv. Funct. Mater. 2021, 31, 2101887.

[58] N. Lee , S. Kim , G. Park , Materials 2019, 12, 3591. 31683748 10.3390/ma12213591 PMC6862661

[59] I. Papanikolaou , C. Litina , A. Zomorodian , A. Al‐Tabbaa , Materials 2020, 13, 5833. 33371389 10.3390/ma13245833 PMC7767458

[60] J. Xu , W. Yao , R. Wang , Cem. Concr. Compos. 2011, 33, 444.

[61] N. Xu , L. Jiang , H. Zhou , H. Chu , P. Jiang , Sci. Ed. 2021, 36, 804.

[62] D. Yuan , W. Jiang , Z. Tong , J. Gao , J. Xiao , W. Ye , Materials (Basel) 2019, 12. 10.3390/ma12233868 PMC6926695 31771187

[63] D. Lu , L. P. Ma , J. Zhong , J. Tong , Z. Liu , W. Ren , H. M. Cheng , ACS Nano 2023, 17, 3587. 36745408 10.1021/acsnano.2c10141

[64] S. Gwon , H. Kim , M. Shin , Cem. Concr. Compos. 2023, 137, 104942.

[65] Y. Cao , K. E. Uhrich , J. Bioact. Comp. Polym. 2018, 34, 3.

[66] D. Belaineh , J. W. Andreasen , J. Palisaitis , A. Malti , K. Håkansson , L. Wågberg , X. Crispin , I. Engquist , M. Berggren , ACS Appl. Polym. Mater 2019, 1, 2342.

[67] J. M. Dodda , M. G. Azar , P. Bělský , M. Šlouf , A. Brož , L. Bačáková , J. Kadlec , T. Remiš , Cellulose 2022, 29, 6697.

[68] X. Wang , X. Sun , D. Gan , M. Soubrier , H.‐Y. Chiang , L. Yan , Y. Li , J. Li , S. Yu , Y. Xia , K. Wang , Q. Qin , X. Jiang , L. Han , T. Pan , C. Xie , X. Lu , Matter 2022, 5, 1204.

[69] D. Zhao , Q. Zhang , W. Chen , X. Yi , S. Liu , Q. Wang , Y. Liu , J. Li , X. Li , H. Yu , ACS Appl. Mater. Interfaces 2017, 9, 13213. 28349683 10.1021/acsami.7b01852

[70] P. Wang , J. Liang , W. Tian , K. Zhang , Y. Xia , Sci. China Mater. 2024, 67, 580.

[71] F. Wang , H.‐J. Kim , S. Park , C.‐D. Kee , S.‐J. Kim , I.‐K. Oh , Compos. Sci. Technol. 2016, 128, 33.

[72] S. Uzunçar , N. Özdoğan , M. Ak , Mater. Today Commun. 2021, 26, 101839.

[73] S. Selvam , Y.‐H. Jo , A. Chan , M. Cumming , M. Jordan , R. Khadka , J.‐H. Yim , J. Energy Storage 2024, 96, 112735.

[74] H. Zheng , M. Chen , Y. Sun , B. Zuo , Chem. Eng. J. 2022, 446, 136931.

[75] S. Sultana , N. Ahmad , S. M. Faisal , M. Owais , IET Nanobiotechnol. 2017, 11, 835.

[76] V. Gautam , K. P. Singh , V. L. Yadav , Int. J. Biol. Macromol. 2018, 111, 1124. 29360546 10.1016/j.ijbiomac.2018.01.094

[77] V. Gautam , A. Srivastava , K. P. Singh , V. L. Yadav , Polym. Compos. 2017, 38, 496.

[78] A. M. Youssef , M. S. Hasanin , M. E. El‐Aziz , G. M. Turky , Int. J. Biol. Macromol. 2021, 167, 1435. 33202266 10.1016/j.ijbiomac.2020.11.097

[79] A. A. Anwar , J. Environ. Anal. Chem. 2023, 104, 9454.

[80] N. Contreras‐Pereda , S. Suárez‐García , R. Pfattner , D. Ruiz‐Molina , Mater. Today Chem. 2024, 35, 101855.

[81] J. V. Paulin , F. O. C. , J. Mater. Chem. C 2021, 9, 14514.

[82] S. Lee , T. Kim , S. Kim , H. Park , J. Lee , B. S. Shim , ACS Appl. Polym. Mater 2024, 6, 11205.

[83] K. S. Kim , W. Y. Maeng , S. Kim , G. Lee , M. Hong , G. B. Kim , J. Kim , S. Kim , S. Han , J. Yoo , H. Lee , K. Lee , J. Koo , Mater Today Bio 2023, 18, 100541. 10.1016/j.mtbio.2023.100541 PMC9840151 36647537

[84] S. M. Won , J. Koo , K. E. Crawford , A. D. Mickle , Y. Xue , S. Min , L. A. McIlvried , Y. Yan , S. B. Kim , S. M. Lee , B. H. Kim , H. Jang , M. R. MacEwan , Y. Huang , R. W. Gereau , J. A. Rogers , Adv. Funct. Mater. 2018, 28, 1801819.

[85] M. Motadayen , S. Agarwala , in IEEE Int. Conf. on Flexible and Printable Sensors and Systems (FLEPS), IEEE, Tampere, Finland 2024.

[86] K. S. Kim , J. Yoo , J. S. Shim , Y. I. Ryu , S. Choi , J. Y. Lee , H. M. Lee , J. Koo , S. K. Kang , Adv. Mater. Technol. 2021, 7, 2001297.

[87] Z. Wei , X. Ma , H. Zhao , X. Wu , Q. Guo , ACS Appl. Mater. Interfaces 2022, 14, 33472. 35830227 10.1021/acsami.2c04647

[88] J. Li , H. Xu , Z. Zhang , Y. Hao , H. Wang , X. Huang , Adv. Funct. Mater. 2019, 30, 1905024.

[89] A. Zareei , V. Selvamani , S. Gopalakrishnan , S. Kadian , M. K. Maruthamuthu , Z. He , J. Nguyen , H. Wang , R. Rahimi , Adv. Mater. Technol. 2022, 7, 2101722.

[90] N. Fumeaux , D. Briand , Npj Flex Electron 2023, 7, 14. 38665150 10.1038/s41528-023-00249-0 PMC11041761

[91] C. L. Baumbauer , A. Gopalakrishnan , M. Atreya , G. L. Whiting , A. C. Arias , Adv. Electron. Mater. 2024, 10, 2300658.

[92] L. Yin , H. Cheng , S. Mao , R. Haasch , Y. Liu , X. Xie , S. W. Hwang , H. Jain , S. K. Kang , Y. Su , R. Li , Y. Huang , J. A. Rogers , Adv. Funct. Mater. 2013, 24, 645.

[93] N. T. Kirkland , J. Lespagnol , N. Birbilis , M. P. Staiger , Corros. Sci. 2010, 52, 287.

[94] L. Rossrucker , K. J. J. Mayrhofer , G. S. Frankel , N. Birbilis , J. Electrochem. Soc. 2014, 161, C115.

[95] R. Arbaud , M. Najafi , J. M. Gandarias , M. Lorenzini , U. C. Paul , A. Zych , A. Athanassiou , P. Cataldi , A. Ajoudani , Adv. Mater. Technol. 2024, 9, 2301265.

[96] P. Cataldi , P. Steiner , M. Liu , G. Pinter , A. Athanassiou , C. Kocabas , I. A. Kinloch , M. A. Bissett , Adv. Funct. Mater. 2023, 33, 2301542.

[97] G. Spallanzani , M. Najafi , M. Zahid , E. L. Papadopoulou , L. Ceseracciu , M. Catalano , A. Athanassiou , P. Cataldi , A. Zych , Adv. Sustainable Syst. 2023, 7, 2300220.

[98] C. L. Lam , IEEE SENSORS,IEEE, Baltimore, MD, USA 2013.

[99] A. Kausar , I. Ahmad , M. Maaza , M. Eisa , P. Bocchetta , J. Comp. Sci. 2022, 6, 393.

[100] M. Abulikemu , B. E. Tabrizi , H. M. Mofarah , K. R. Shad , G. E. Jabbour , Flex. Print. Electron. 2023, 8, 024004.

[101] N. Nasr , I. A. K. Shahzad , C. F. Dee , B. Bais , T. Zhao In Carbonous‐Based Optoelectronic Devices, Enhanced Carbon‐Based Materials and Their Applications, (Eds: P.C. Ooi , M. Xie , C.F. Dee ), Wiley‐VCH, Weinheim, 2022.

[102] P. R. de Oliveira , C. Kalinke , J. A. Bonacin , L. H. Marcolino‐Junior , M. F. Bergamini , O. Malaspina , R. C. Nocelli , B. C. Janegitz , Electrochim. Acta 2021, 390, 138876.

[103] D. C. de Souza , L. O. Orzari , P. R. de Oliveira , C. Kalinke , J. A. Bonacin , O. Malaspina , R. C. F. Nocelli , B. C. Janegitz , Food Anal. Methods 2020, 14, 606.

[104] M. Shadabfar , M. Ehsani , H. A. Khonakdar , M. Abdouss , T. Ameri , Cellulose 2022, 30, 1759.

[105] X. Liao , Z. Zhang , Q. Liao , Q. Liang , Y. Ou , M. Xu , M. Li , G. Zhang , Y. Zhang , Nanoscale 2016, 8, 13025. 27314505 10.1039/c6nr02172g

[106] M. Shadabfar , M. Ehsani , H. A. Khonakdar , M. Abdouss , Polym. Compos. 2023, 45, 475.

[107] P. Jezowski , P. L. Kowalczewski , Polymers 2019, 11, 1648. 31614451 10.3390/polym11101648 PMC6836256

[108] P. Ruschhaupt , A. Varzi , S. Passerini , ChemSusChem 2020, 13, 763. 31815362 10.1002/cssc.201902863 PMC7065209

[109] P. Cataldi , I. S. Bayer , F. Bonaccorso , V. Pellegrini , A. Athanassiou , R. Cingolani , Adv. Electron. Mater. 2015, 1, 1500224.

[110] L. A. Romero‐Cano , A. I. Zárate‐Guzmán , F. Carrasco‐Marín , L. V. González‐Gutiérrez , J. Electroanal. Chem. 2019, 837, 22. 10.1016/j.chemosphere.2019.02.101 30851521

[111] T. Tu , B. Liang , Q. Cao , L. Fang , Q. Zhu , Y. Cai , X. Ye , RSC Adv. 2020, 10, 7241. 35493906 10.1039/c9ra09847j PMC9049791

[112] P. Cataldi , L. Lamanna , C. Bertei , F. Arena , P. Rossi , M. Liu , F. Di Fonzo , D. G. Papageorgiou , A. Luzio , M. Caironi , Adv. Funct. Mater. 2022, 32, 2113417.

[113] G. V. C.‐P. N. , P. Cataldi , V. F. Annese , G. Coco , A. Athanassiou , A. Luzio , Wiley Small Sci. 2024, 5, 2400373. 10.1002/smsc.202400373 PMC11935078 40212641

[114] A. Zych , M. Contardi , C. Rinaldi , V. Scribano , V. Isa , D. Kossyvaki , J. Gobbato , L. Ceseracciu , S. Lavorano , P. Galli , A. Athanassiou , S. Montano , Adv. Sustainable Syst. 2024, 8, 2400110.

[115] M. A. Meier , J. O. Metzger , U. S. Schubert , Chem. Soc. Rev. 2007, 36, 1788. 18213986 10.1039/b703294c

[116] Q. Tang , Y. Chen , H. Gao , Q. Li , Z. Xi , L. Zhao , C. Peng , L. Li , In Bio‐Based Epoxy Resin from Epoxidized Soybean Oil, Soybean‐Biomass, Yield and Productivity, (Eds: M. Kasai ), IntechOpen, London, United Kingdom, 2018.

[117] D. Behera , A. K. Banthia , J. Appl. Polym. Sci. 2008, 109, 2583.

[118] P. Cataldi , A. Athanassiou , I. Bayer , Appl. Sci. 2018, 8, 1438.

[119] P. Cataldi , M. Liu , M. Bissett , I. A. Kinloch , Adv. Mater. Technol. 2022, 7, 2200025.

[120] V. Orts Mercadillo , K. C. Chan , M. Caironi , A. Athanassiou , I. A. Kinloch , M. Bissett , P. Cataldi , Adv. Funct. Mater. 2022, 32, 2204772.

[121] C. Mendes‐Felipe , R. Cofano , A. Garcia , M. Sangermano , S. Lanceros‐Mendez , Addit. Manuf. 2023, 78, 103867.

[122] V. F. Annese , P. Cataldi , V. Galli , G. Coco , J. P. Damasceno , A. Keller , Y. Kumaresan , P. Rossi , I. K. Ilic , B. Kwak , L. T. Kubota , A. Athanassiou , J. Rossiter , D. Floreano , M. Caironi , Adv. Sensor Res. 2023, 3, 2300150.

[123] J. Erkmen , H. I. Yavuz , E. Kavci , M. Sari , Constr. Build. Mater. 2020, 255, 119357.

[124] P. A. Kumar , Int. J. Sci. Res. Publ. 2012, 2, https://www.ijsrp.org/research‐paper‐publishing‐jul‐2012.php.

[125] N. Elbers , C. K. Ranaweera , M. Ionescu , X. Wan , P. K. Kahol , R. K. Gupta , J. Renew. Mater. 2017, 5, 74.

[126] M. Kermani , M. Farzaneh , R. Gagnon , Cold Reg. Sci. Technol. 2007, 49, 195.

[127] L. Ma , J. Wu , M. Wang , L. Dong , H. Wei , Engineering Geology 2020, 272, 105615.

[128] X. Wei , L. Xiao , Z. Li , Constr. Build. Mater. 2012, 31, 341.

[129] P. Cataldi , M. Cassinelli , J. A. Heredia‐Guerrero , S. Guzman‐Puyol , S. Naderizadeh , A. Athanassiou , M. Caironi , Adv. Funct. Mater. 2019, 30, 1907301.

[130] G. P. Kotchey , B. L. Allen , H. Vedala , N. Yanamala , A. A. Kapralov , Y. Y. Tyurina , J. Klein‐Seetharaman , V. E. Kagan , A. Star , ACS Nano 2011, 5, 2098. 21344859 10.1021/nn103265h PMC3062704

[131] R. Kurapati , F. Bonachera , J. Russier , A. R. Sureshbabu , C. Ménard‐Moyon , K. Kostarelos , A. Bianco , 2D Mater. 2017, 5, 015020.

[132] R. Kurapati , J. Russier , M. A. Squillaci , E. Treossi , C. Menard‐Moyon , A. E. Del Rio‐Castillo , E. Vazquez , P. Samori , V. Palermo , A. Bianco , Small 2015, 11, 3985. 25959808 10.1002/smll.201500038

[133] G. Lalwani , W. Xing , B. Sitharaman , J. Mater. Chem. B 2014, 2, 6354. 25215188 10.1039/C4TB00976B PMC4157692

[134] B. Fadeel , C. Bussy , S. Merino , E. Vazquez , E. Flahaut , F. Mouchet , L. Evariste , L. Gauthier , A. J. Koivisto , U. Vogel , C. Martin , L. G. Delogu , T. Buerki‐Thurnherr , P. Wick , D. Beloin‐Saint‐Pierre , R. Hischier , M. Pelin , F. Candotto Carniel , M. Tretiach , F. Cesca , F. Benfenati , D. Scaini , L. Ballerini , K. Kostarelos , M. Prato , A. Bianco , ACS Nano 2018, 12, 10582. 30387986 10.1021/acsnano.8b04758

[135] B. Huang , T. Yuan , Y. Liang , Y. Guo , X. Yuan , W. Zhou , H. Huang , S. Liu , AEHM 2020, 23, 332.

[136] W. H. Hilbertz , T. J. Goreau , Method of enhancing the growth of aquatic organisms, and structures created thereby United States of America Patent 1996.

[137] R. C. Dorf , The Electronics Handbook, Vol. 34, Taylor & Francis Group, Milton Park, in Oxfordshire, 2005.

[138] K. H. Amir , A. Jabir , M. Fawzi , H. A.l‐H. Qusay , G. A. Al‐Najar , A. A. Jassim , SJAR 2024, 11, 82.

[139] D. Natasasmita , D. P. Wijayanti , C. A. Suryono , J. Aquac. Mar. Biol. 2016, 4, 11.

[140] J. N. Awaya , Bachelor of Science in Global Environmental Science, Hawai Institute of Marine Biology, United States, 2023.

[141] I. S. S. Knapp , Z. H. Forsman , A. Greene , E. C. Johnston , C. E. Bardin , N. Chan , C. Wolke , D. Gulko , R. J. Toonen , PeerJ 2022, 10, 13653. 10.7717/peerj.13653 PMC9302430 35873907

[142] G. Herrera , A. Good , A. Hirota , C. Razal , N. Gaertner , J. Sefcik , J. Gilbert , K. Bahr , AJUR 2023, 20, 27.

[143] U. E. Siebeck , N. J. Marshall , A. Klüter , Coral Reefs 2006, 25, 453.

[144] M. Contardi , S. Montano , G. Liguori , J. A. Heredia‐Guerrero , P. Galli , A. Athanassiou , I. S. Bayer , Sci. Rep. 2020, 10, 988. 31969660 10.1038/s41598-020-57980-1 PMC6976594

[145] M. Contardi , M. Fadda , V. Isa , Y. D. Louis , A. Madaschi , S. Vencato , E. Montalbetti , L. Bertolacci , L. Ceseracciu , D. Seveso , S. Lavorano , P. Galli , A. Athanassiou , S. Montano , ACS Appl. Mater. Interfaces 2023, 15, 33916. 37376819 10.1021/acsami.3c01166 PMC10360034

[146] M. Contardi , S. Montano , P. Galli , G. Mazzon , A. Mah'd Moh'd Ayyoub , D. Seveso , F. Saliu , D. Maggioni , A. Athanassiou , I. S. Bayer , Adv. Sustainable Syst. 2021, 5, 2100089.

[147] J. Liu , B. Ju , W. Xie , H. Yu , H. Xiao , S. Dong , W. Yang , Materials 2021, 14, 5871. 34640267 10.3390/ma14195871 PMC8510195

[148] F. C. Walsh , Transactions of the IMF 1991, 69, 107.

[149] EZIL , Metal Strength Chart: A Detailed Guide To Metal Strengths https://eziil.com/steel‐strengths (accessed: June 2025).

[150] I. Matus Valenzuela , J. Góis , P. Vaz‐Pires , J. Lino Alves , ACS Sustainable Chem. Eng. 2024, 12, 13721.

[151] M. V. Ilse , J. Góis , P. Vaz Pires , L. A. Jorge , in Materials Design and Applications V, Vol. 212, Springer, Berlin, 2024.

