# Supplementary material for: Active Biopaste for Coral Reef Restoration
Source: Adv Mater. 2025 Jul 4;37(38):2502078. doi: 10.1002/adma.202502078 (PMC12464626; doi:10.1002/adma.202502078)
Supplement: Supplementary file 1 — Supporting Information [file ADMA-37-2502078-s001.docx]

Supporting Information

Active Biopaste for Coral Reef Restoration

*Gabriele Corigliano, Valerio Isa, Valerio Francesco Annese, Camilla Rinaldi, Maria Summa, Rosalia Bertorelli, Paolo Galli, Silvia Lavorano, Mario Caironi, Marco Contardi*, Pietro Cataldi*, Simone Montano*^, +^, Athanassia Athanassiou*^, +^*

**Attenuated total reflection-Fourier-transform infrared spectroscopy** (**ATR-FTIR**)

**Figure S 1** showcases the ATR-FTIR spectra for ESOA, components A and B, and the AB paste. The spectra were baseline corrected to allow better comparison between traces. The red vertical lines highlight the acrylate peak, 1637 cm^-1^, which is represented enlarged in **Figure S 2** for A, B and AB. The decrease in magnitude of the peak from A and B to AB demonstrates that crosslinking has occurred. Specifically, for the ratio between peak areas A2 and A1 a reduction after crosslinking of 69.4% was observed and the ratio between peak areas P2 and P1 showed a reduction of 63.4%.


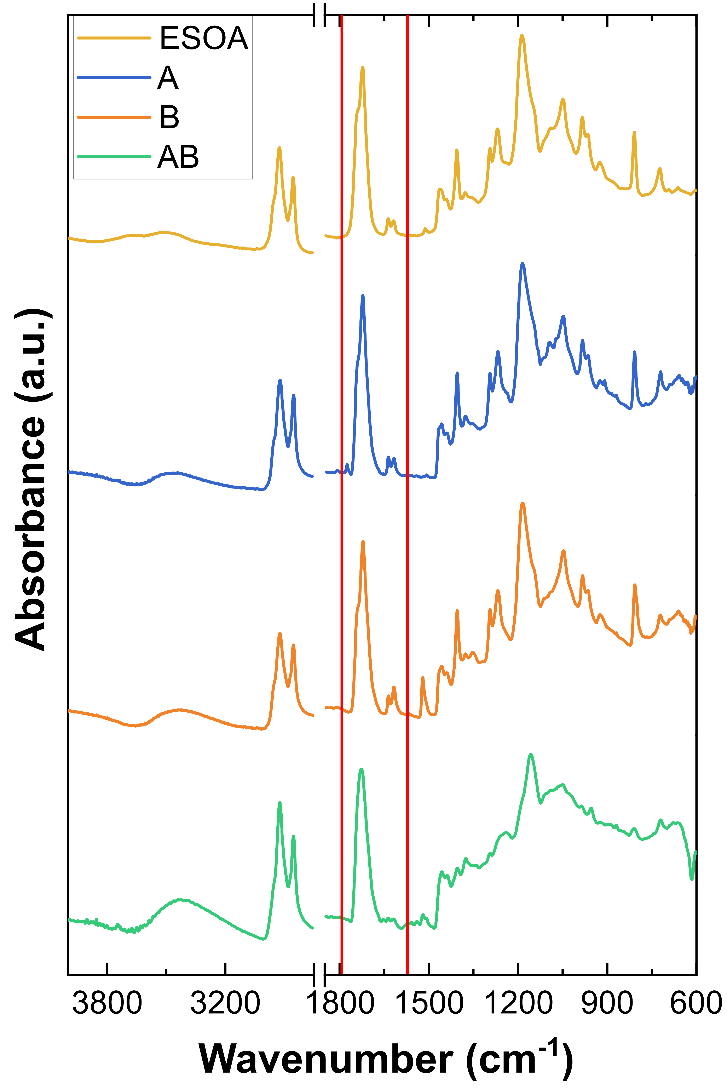


**Figure S 1:** ATR**-**FTIR spectra of pure ESOA as well as components A, B, and the AB paste.


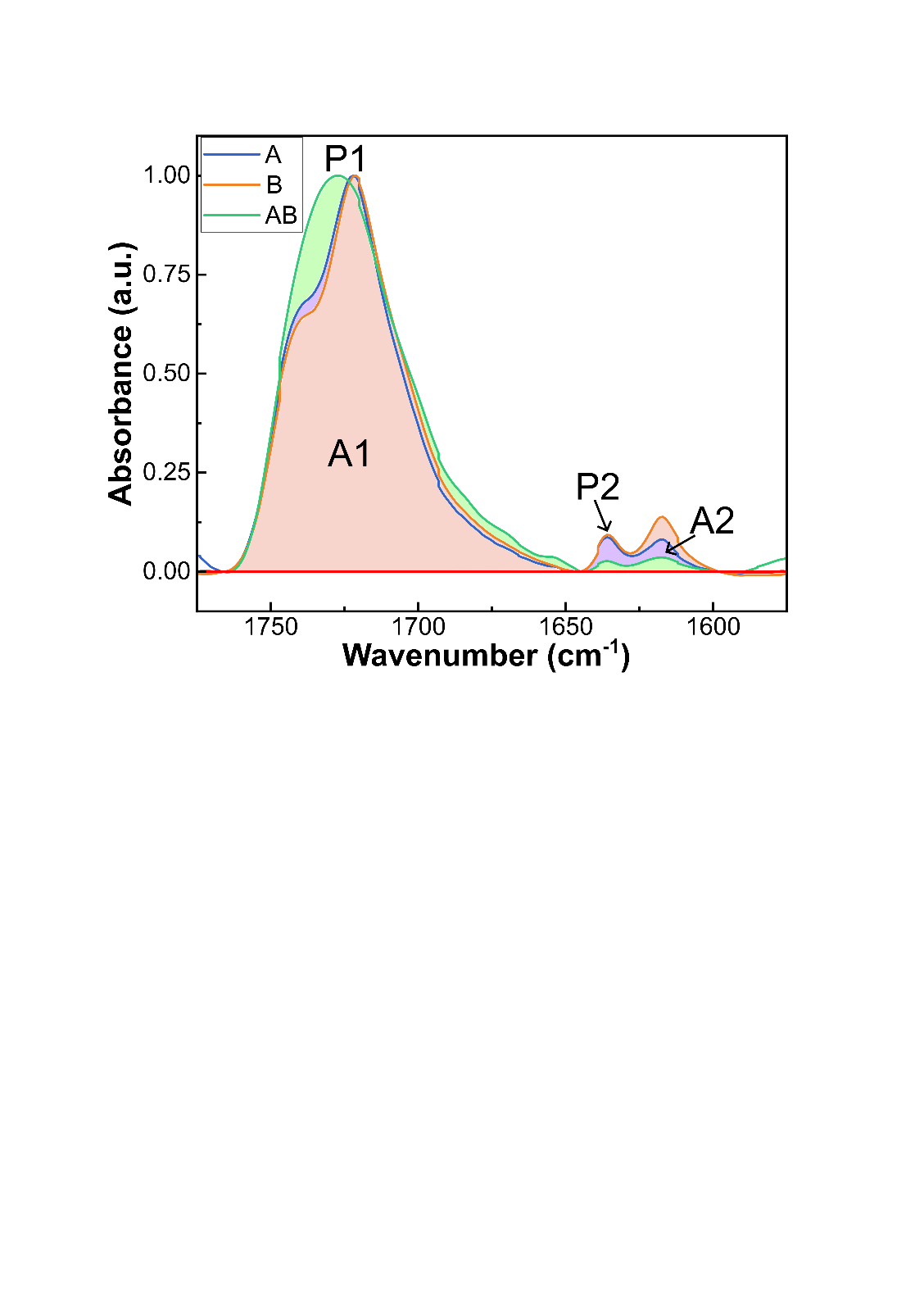


**Figure S 2:** ATR-FTIR spectra expanded to display peaks for the acrylate group at 1637 cm^-1^.

**Impedance phases**


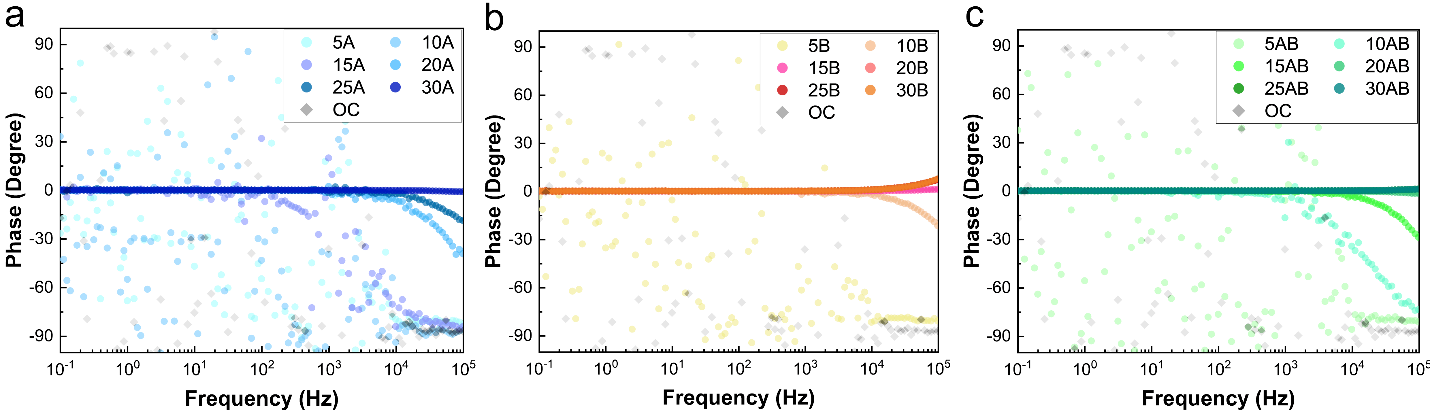


**Figure S 3:** a, b, c: Impedances phases for components A, B and the AB paste, respectively, at increasing GnPs loading percentages. OC=open circuit.

**Resistance variation in seawater**

**
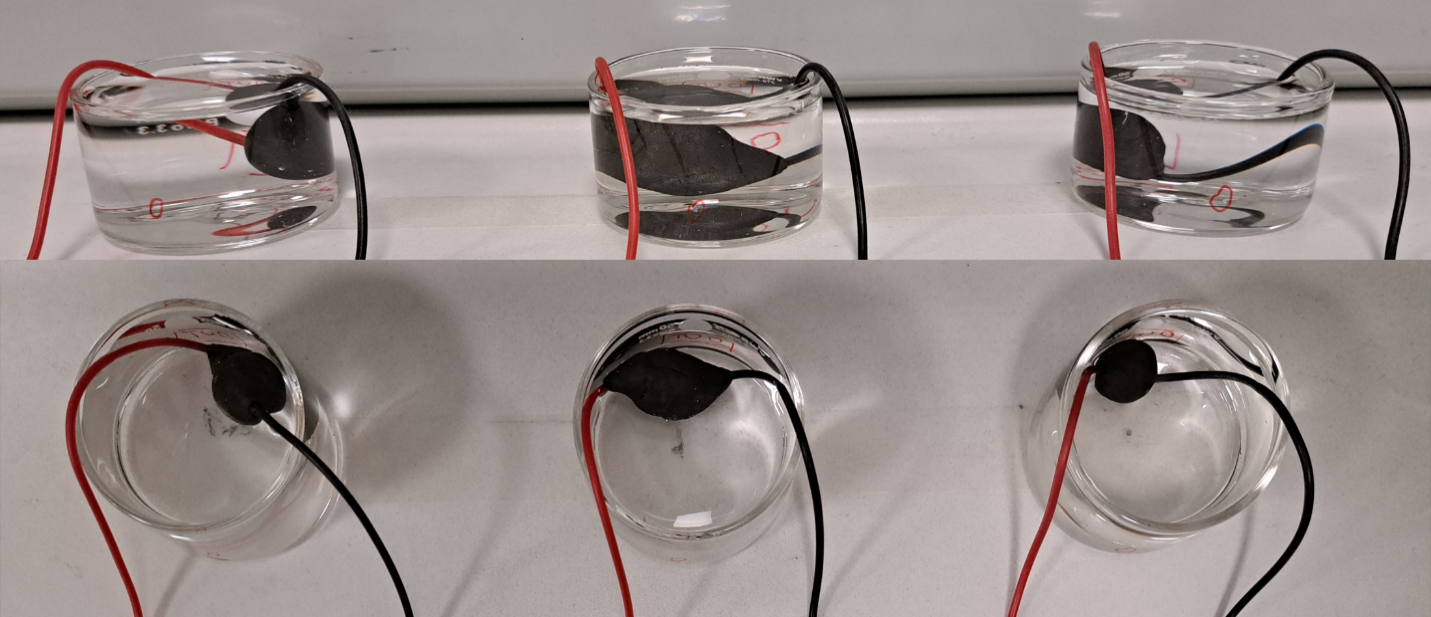
**

**Figure S 4:** Pictures showcasing crosslinked AB pastes submerged in seawater with wires immersed in it. This setup was employed to study the resistance variation of the pastes during prolonged immersion in seawater (30 days).

**Water contact angle (WCA)**


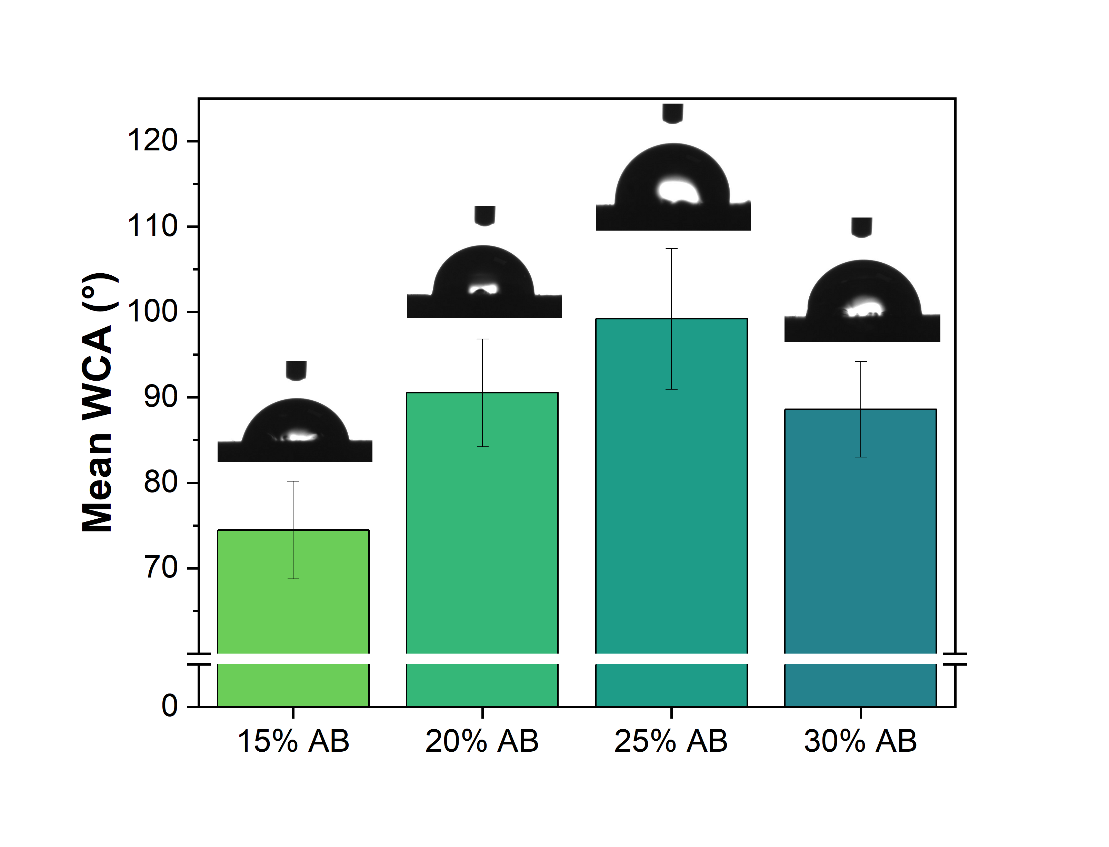


**Figure S 5:**WCA for paste AB with GnPs loading from 15 to 30% by wt.

**Release in water**

Given that even minuscule amounts of GnPs dispersed in water alter the liquid's appearance from transparent to black, the transmittance of the water housing the crosslinked paste changed after several weeks of incubation was monitored to assess possible GnPs release. Seawater was employed to account for any additional erosion caused by salts. To minimize the proliferation of algae and bacteria contained in the seawater, it was sterilized under UV light, and the samples were maintained at 4 °C. Algae proliferation was undesirable because it leads to a shift in water color that would yield variations in its transmittance unrelated to GnPs release. The samples were positioned in 10 mL falcons. Controls (only seawater) and tests (paste immersed in seawater) were kept closed until the day of the measurement. Based on the findings depicted in **Figure S 6**, it can be stated that over the time period examined, there was no significant variation in the transmittance of seawater between the control and test conditions. This leads to the conclusion that no observable macroscopic release of GnPs into the water occurred.


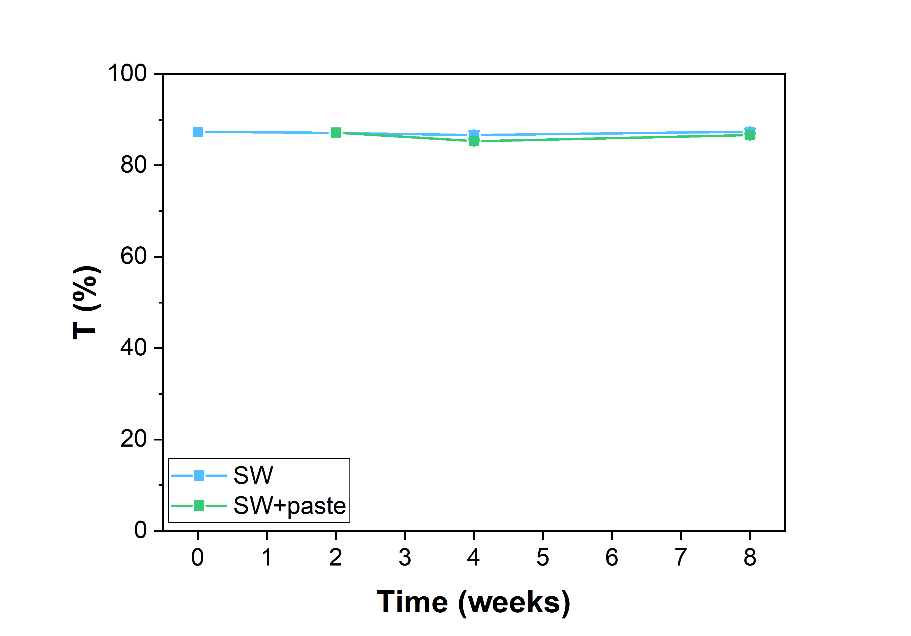


**Figure S 6:** Transmittance percentages at 600 nm for both seawater and seawater containing the AB paste in immersion. Results span over 8 weeks.

**Impedance in water**

In **Figure S 7**, the results for the analysis of the impedance of components A, B, and paste AB when immersed in water are reported. The pastes were tested when immersed in MilliQ, tap, and seawater. Measurements were done before and during immersion. **Figure S 7a 7b**, and **7c** showcase the impedance moduli of the materials while **Figure S 7d, 7e, 7f** display the corresponding phases. **Figure S 7a** and **7d** represent the behavior of the materials when immersed in MilliQ (MQ) water. **Figure S 7b** and **7e** contains measurements for their behavior when immersed in tap water. **Figure S 7c** and **7d** show their behavior when immersed in seawater. The notation (air) indicates that the measurement was done before immersion, while the notations MQW, TW, and SW mean that the measurements were done during immersion in MQ, tap, and seawater, respectively. The impedances of MQ, tap, and seawater are additionally plotted as a comparison. For the experiment, the wires were encapsulated within the materials to try to minimize the effect of water impedance on the measurements. It is interesting to observe that the impedances moduli and phases of component B and paste AB do not vary when immersed in water. This outcome is the same for all water types, indicating that the pastes effectively insulate the wires from water. Such water resistance is highly advantageous given the intended application of these materials. For component A, it was observed that the impedance does not vary during immersion in MQ water, but it does when samples are immersed in tap and seawater. This means that in the two latter conditions component A fails to guarantee complete water insulation. One potential explanation could be the more brittle nature of component A in comparison to component B and paste AB. The increased brittleness of component A could compromise its structural integrity, making it less adept at forming an effective barrier against water. Nevertheless, the results for the crosslinked paste AB, which is final material to be employed underwater, are excellent.

Components A and B and the paste AB were positioned on lab glass slides and measured their impedances before and during immersion in seawater (**Figure S 8**)**.** In this setup, the wires were exposed to seawater, so the impedance measurements accounted for the conductivity of seawater. In this configuration, water was exactly parallel with the biopaste. Consequently, the electrical field path through the water exhibited minor impedance compared to when the wires were embedded within the pastes. The decrease in impedance moduli was observed in A, B, and AB, but was more evident in the materials with higher impedance, i.e., component A. Notably, the behavior of component B remained predominantly resistive even during immersion in seawater. This conclusion was reached by observing that the impedance of B measured in air was not lower than the one of seawater even at high frequencies.


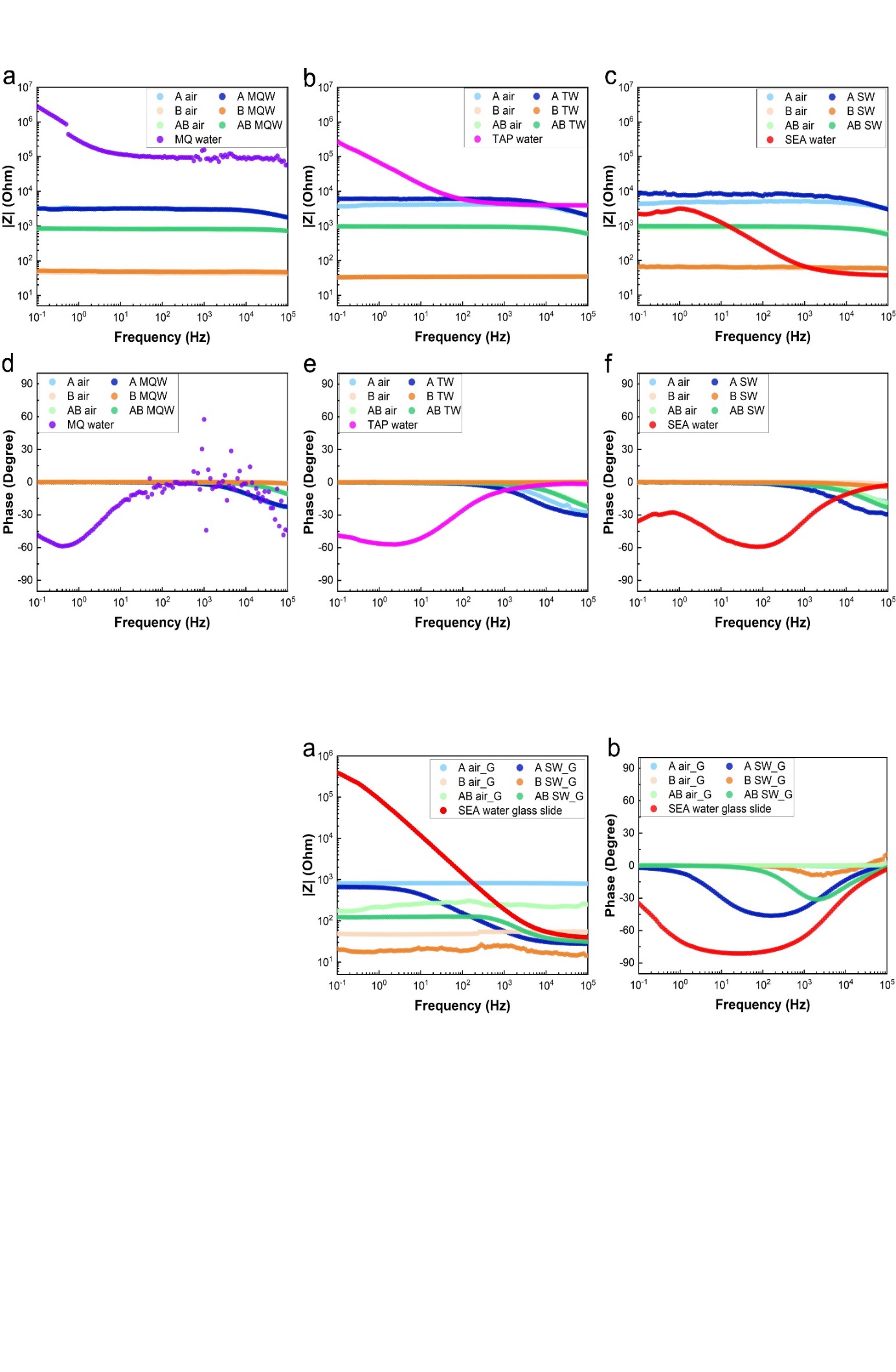


**Figure S 7:** a, b, c: Impedance moduli of components A, B and paste AB in MQ, tap, and seawater, respectively. d, e, f: The corresponding phases. For the measurements wires were encapsulated in the pastes.


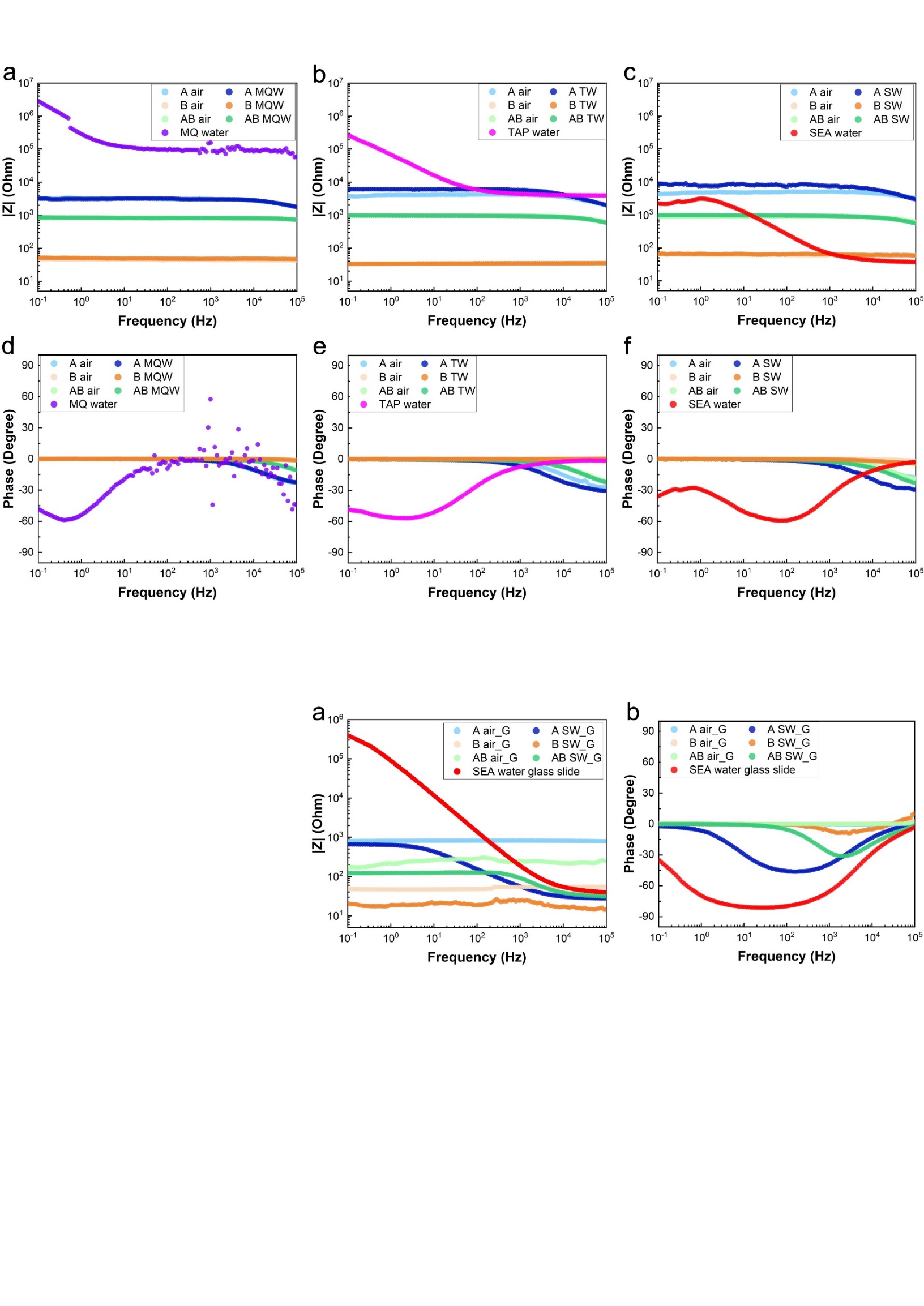


**Figure S 8:** a, b: Modulus and phase of the impedances of components A, B, and paste AB in MQ, tap, and seawater. The impedances were measured on a lab glass slide; therefore the electrical contacts were exposed to water.

**Rheology**

**Figure S 9** illustrates the results of the rheological measurements conducted on the pastes, encompassing an amplitude sweep test (a-b), from 0.001 to 10% of shear strain at 10 rad/s, a frequency sweep test (c-d) from 0.1 to 100 rad/s at a percentual shear strain of 0.05%, and a time sweep test (e-f) with a constant angular frequency of 10 rad/s and a constant shear strain of 0.05%. All the tests were performed at 28 °C to simulate the water temperature that is usually found in tropical environments. In **Figure S 9a, 9c** and **9e** the Storage (G′) and Loss (G′′) moduli are reported, while in **Figure S 9b, 9d** and **9f** the complex viscosity (η*) is reported. The complex viscosity is a measure of the total resistance to flow as a function of angular frequency and is defined as $\eta^{*}=G^{*}/\omega$, where G* is the vectorial sum of G′ and G′′ and ω is the angular frequency. The amplitude sweep test was instrumental in finding the Linear Viscoelastic Region, defined as the range in which the sample's structure remains intact under testing conditions. This region is found at low shear strain, <0.05%, in the left portion of **Figure S 9a** and **9b** where G′ and G′′ are constant and hence independent to variations shear strain. At high shear strain G′ and dropped and G′′ surpassed G′ representing breakdown of the sample structure. Analyzing the frequency sweep test, it is possible to observe that components A, with varying initiator loadings, and B exhibited solid-gel-like behavior. This is evidenced by G′ being consistently higher and parallel to G′′ across the evaluated frequency range, resulting in a phase angle below 45°. Components A and B demonstrated shear-thinning behavior, indicated by a decrease in complex viscosity with increasing shear rates, i.e., increasing angular frequency. This behavior is evident in the characteristic linear region of the log-log graph shown in **Figure S 9d.**

***
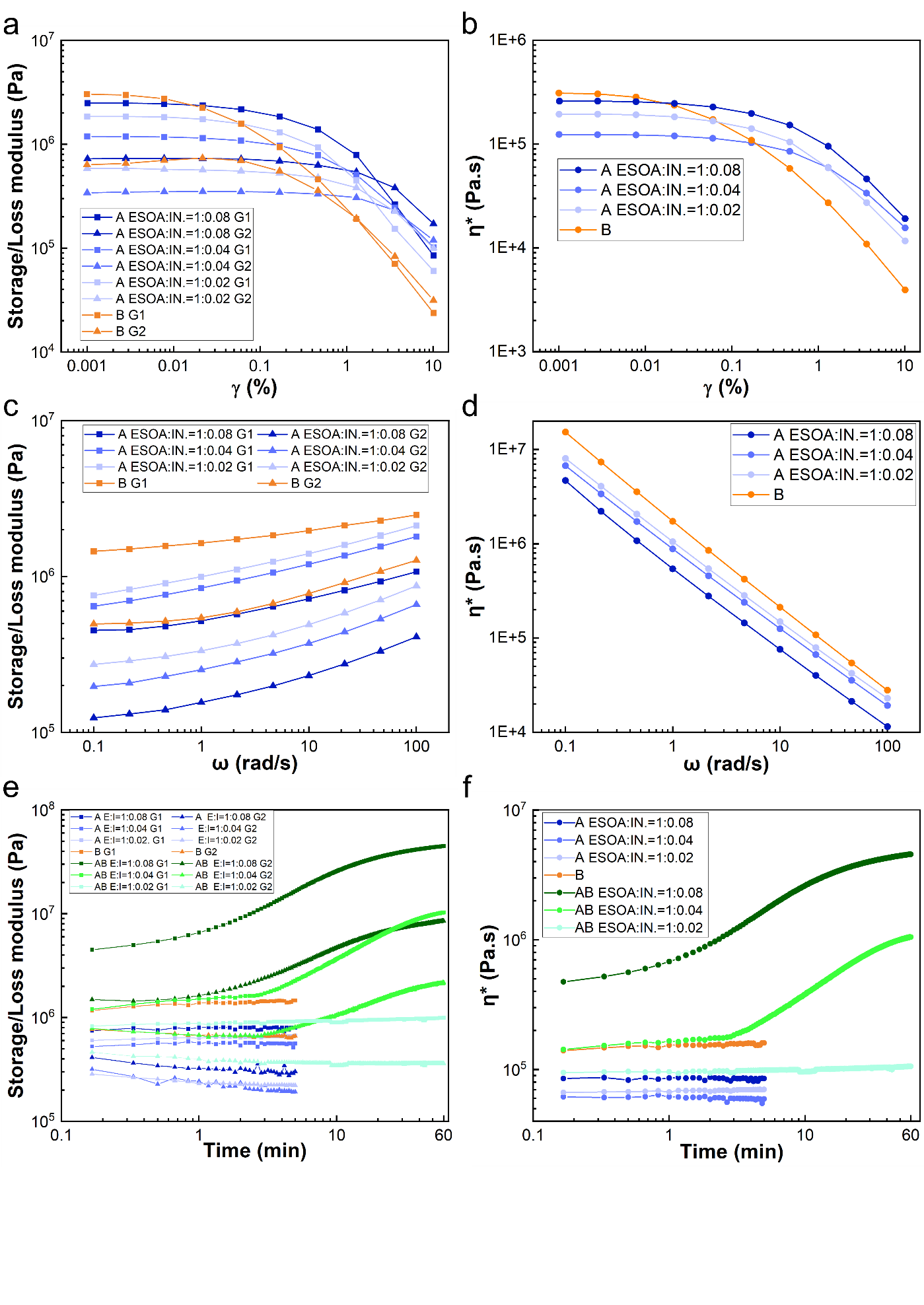
***

**Figure S 9**: a, b: Amplitude sweep test. c, d: Frequency sweep test. e, f: Time sweep test. a, c, e: Storage and loss modulus as function of percentual shear strain, angular frequency, and time, respectively. b, d, f: complex viscosity as function of percentual shear strain, angular frequency, and time, respectively.

**Compressive test**

*
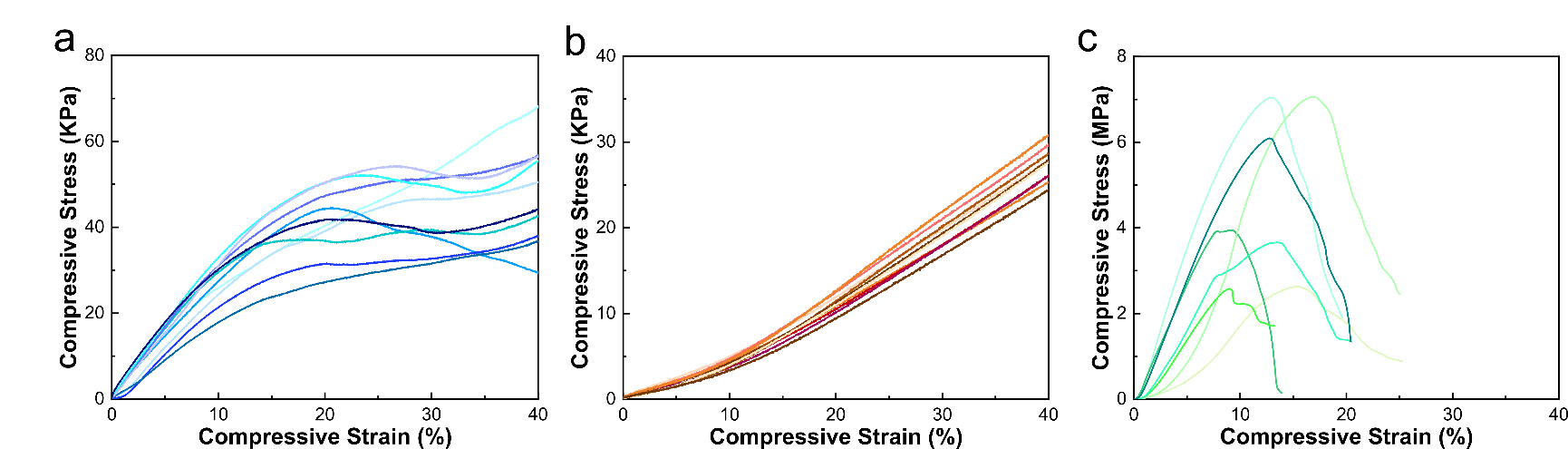
*

**Figure S 10:** a, b, c: Compressive stress-strain curves for components A, B and paste AB, respectively.

**Figure S 10** displays all the compressive tests performed on the samples. The results are more consistent for component A and B, while paste AB has a higher standard deviation.

**Biodegradability test (Biochemical Oxygen Demand)**


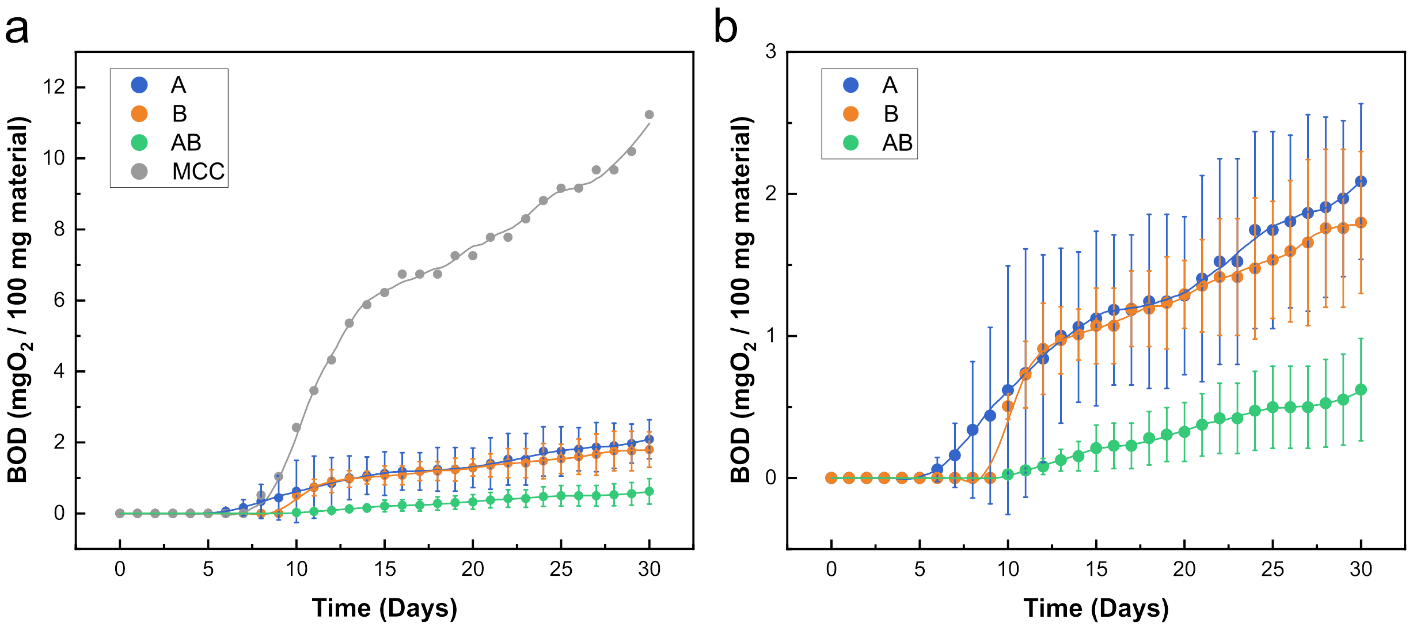


**Figure S 11:** Biochemical Oxygen Demand for components A, B and paste AB. a: the results alongside those for microcrystalline cellulose (MCC), which works as a control. b: a zoom of the conductive pastes. For both MCC and the paste, a low-pass filtered curve of the data is shown.

**Biocompatibility**

**Figure S 12** presents the biocompatibility test results for the pastes on keratinocytes and fibroblasts at concentrations of 0.5, 1, 5, and 10 mg/mL. The results were consistently satisfactory at both 24 and 48 hours across all concentrations, with the relative cell viability exceeding 70% in all measured instances.

**Figure S 14** illustrates the growth of a coral attached to a rod using the conductive bicomponent; showcasing its progression at three months, six months and 24 months after attachment (from top to bottom). The red arrows highlight how the coral has grown directly onto the paste, signifying that it has encountered a good substrate for its development. The coral's growth enhancement is evident spatially on the substrate between the two timepoints. This result is very promising since it implies that the paste can be used for coral attachment onto the reef and ensures its biocompatibility with corals


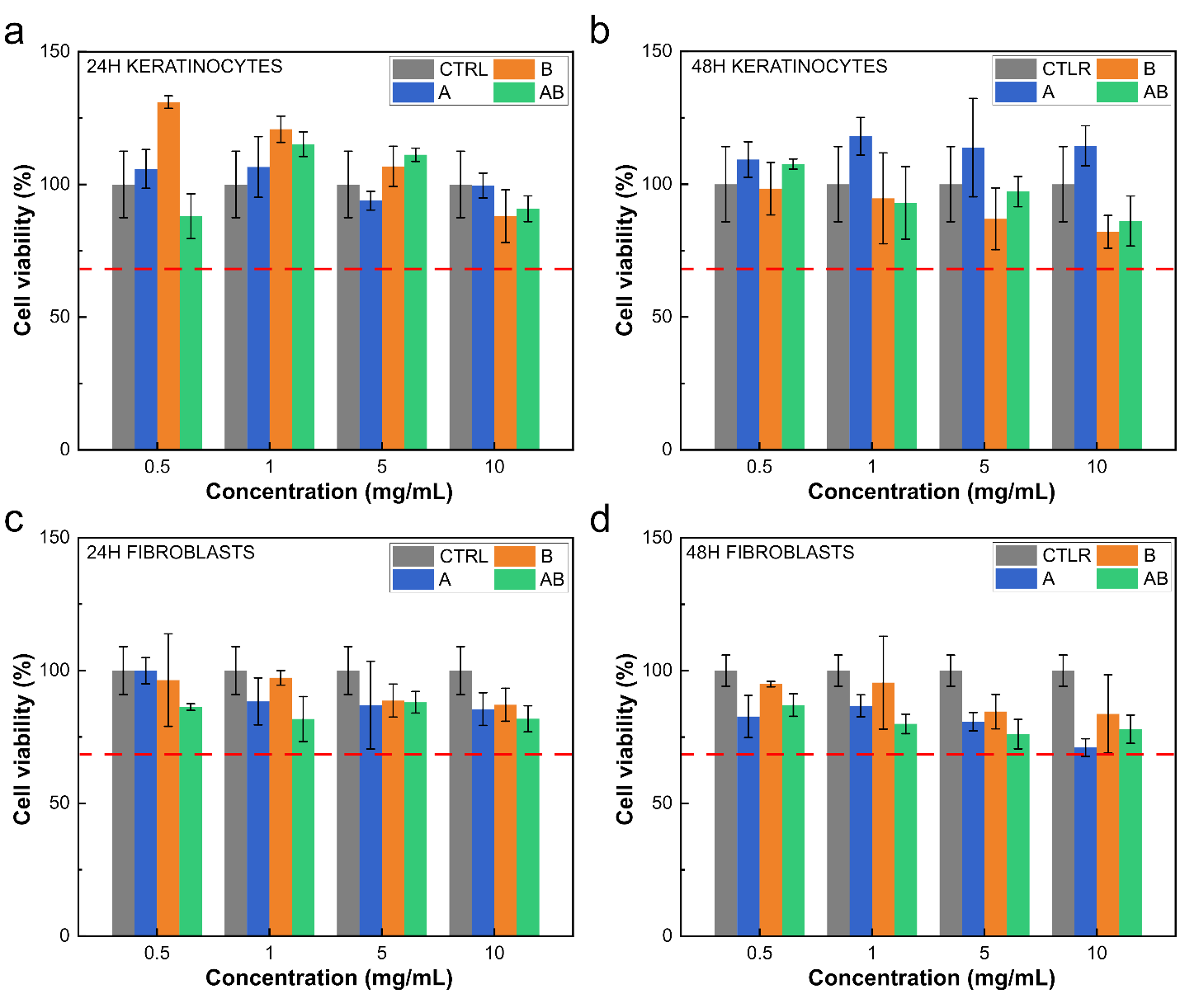


**Figure S 12:** a-b: Biocompatibility results for keratinocytes exposed to components A, B and paste AB at different concentrations over 24h (a) and 48h (b) exposure times. CTRL=control. c-d: Biocompatibility results for fibroblasts at 24h (c) and 48h (d). Red dotted lines indicate the 70% cell viability level above which a material is considered biocompatible according to ISO10993-5 guidelines.


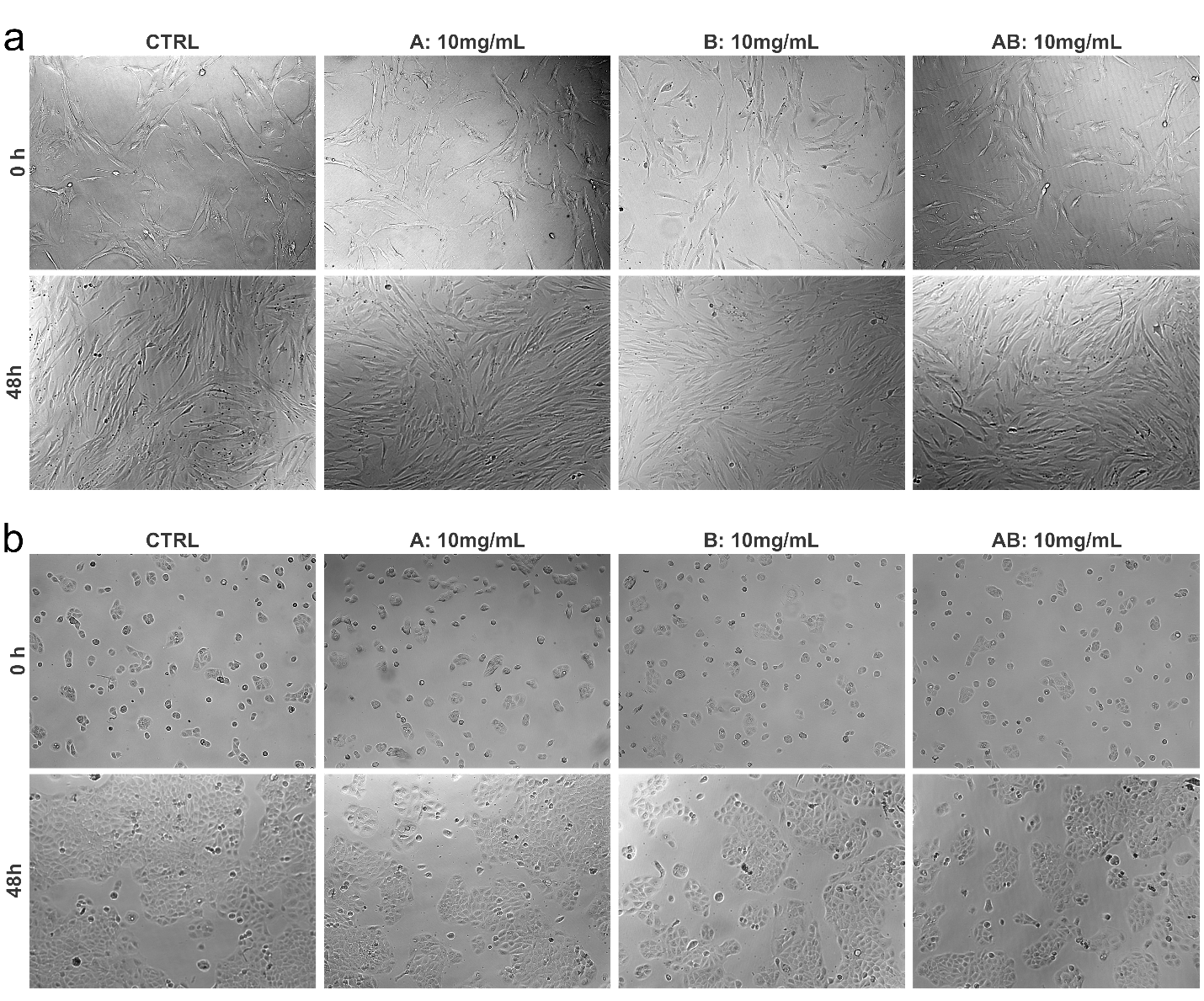


**Figure S 13:** a-b: Pictures of fibroblasts and keratinocytes, respectively, during cytotoxicity assay at 0 and 48 hours for the control (CTRL), components A and B, and AB paste at the concentration of 10 mg/mL.


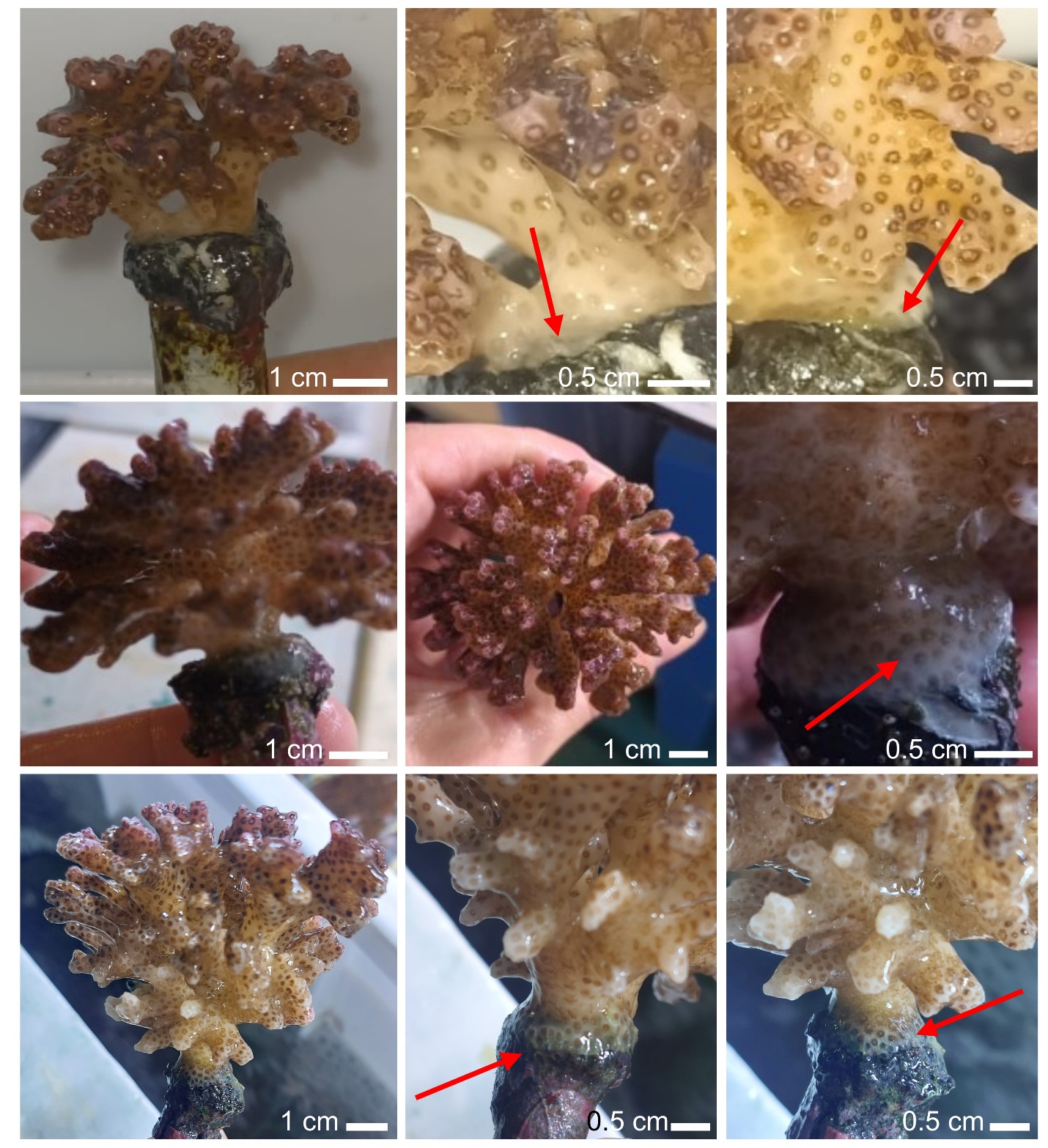


**Figure S 14:** Growth of *Pocillopora* on conductive underwater paste after three months (upper row), six months (middle row), and 24 months (last row).

**Mineral Accretion Technology (MAT) experiments: Parameter determination**

For the success of electrochemical mineral accretion, water electrolysis is needed. Hence a difference of potential (ΔV) higher than 1.229 V, at which water electrolysis occurs, has to be maintained between the anode and the cathode. The best ΔV to promote mineral accretion, as indicated in the Biorock patent, is between 3 to 12 V.

As preliminary tests, seawater from the Genova Aquarium was brought in the lab to assess under which conditions the necessary ΔV was maintained. A ΔV of 12 V between the electrodes and a current of 5 mA at 0 Hz was set. The choice for this current magnitude was related to safety concerns related to electricity hazards. For AC currents at 60 Hz the perception threshold for an electrical current is 1 mA, while for DC currents is at 5 mA.^[137]^ The let go currents are instead 10 mA in AC at 60 Hz and 75 mA in DC.

When two stainless steel electrodes were tested, the ΔV between them was not maintained since stainless steel has a remarkably high conductivity and the current needed to maintain it is very high. The same occurred if only the anode was metallic. Thus, experiments with various setups using the conductive paste for both the cathode and anode were conducted. In each case, a ΔV > 1.229 V was maintained even when adjusting the amount of paste forming the anode to alter the electrode’s resistance. This outcome resulted from the higher resistivity of the conductive paste compared to stainless steel, preventing a "short circuit" between the electrodes and enabling the maintenance of ΔV even at low currents.

**Figure S 15a** illustrates the set up employed for the experiments in the lab where the cathode was the conductive paste in the shape of a square while the anode was a ball of the paste at varying sizes. Both electrodes were submerged in seawater. **Figure S 15b, 15c** and **15d** display the ΔV sustained by the AC-DC converter for currents of 4 mA, 10 mA, and 50 mA, given a set ΔV of 12 V. In each case ΔV is higher than the desired 1.229 V, however, it is clear that at higher currents the converter keeps the ΔV between the electrodes more easily. In **Figure S 15e, 15f** and **15g,** different relative configurations of anode and cathode are displayed. The anode was positioned at increasing distances from the cathode. Interestingly, the ΔV between the electrodes remained constant despite moving the anode, likely due to internal feedback of the AC-DC converter.


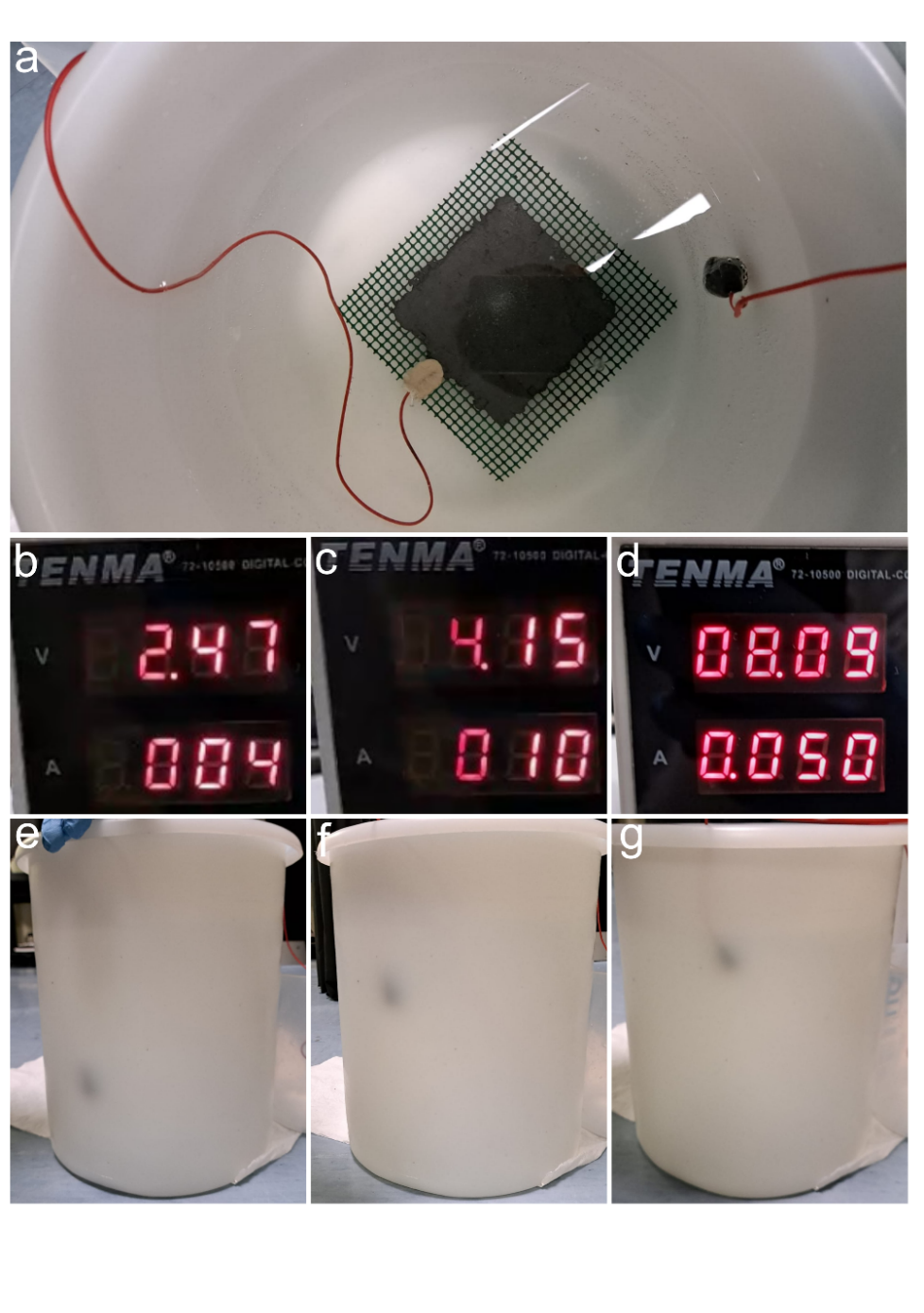


**Figure S 15**: Experiments in the lab with seawater from the Genoa Aquarium. a: Experimental setup. b, c, d: ΔV maintained by the AC-DC converter at different currents when a ΔV=12V was set. e, f, g: Different relative positioning of the anode (black ball at different heights) and the cathode (on the bottom of the tank).

A stability test was then performed for 5 hours in the lab. The setup is shown in **Figure S 16a**. The anode and cathode built with conductive paste were positioned one beside the other in seawater for 5 hours. After building potential and later stabilization, no major modifications in ΔV were observed for the timeframe considered. The ΔV sustained with a 4 mA current is shown in **Figure S 16b**. The results demonstrate effective, long-lasting insulation of the electrodes. Electrolysis is evident from the formation of small gas bubbles on the electrode surfaces.

Given the acidic pH at the anode, detrimental to coral growth, combined with the fact that the experimental setup is positioned in a confined space,^[150-151]^ careful positioning of the anode and cathode relative to water flow was crucial. Optimal placement involves positioning the anode not too distant from the cathode and downstream. In the aquarium setup, **Figure S 17**, this arrangement was maintained for every experiment.

To assess any negative impact on the cathode from anode reactions, the pH variations throughout a day-night cycle were monitored with a Multi-parameter Water Quality Analyzer, BEP-WQ2000. In daytime, water flows from inlet to outlet while at night there was only internal circulation. pH measurements were as follows:

- Day before setup: 7.87
- Day after setup:
  - At anode: 7.86
  - At cathode: 7.86
- pH after night:
  - At anode: 8.15
  - At cathode: 8.17

These results were promising, as optimal pH for coral growth falls between 7.8 and 8.2.


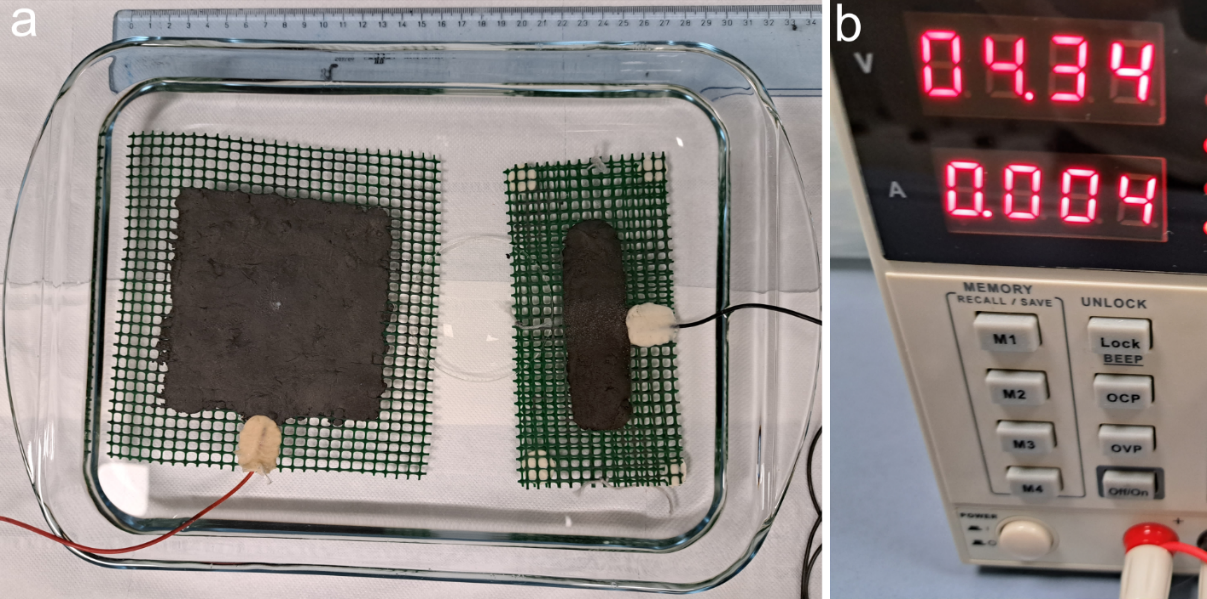


**Figure S 16:** Stability test for the electrodes in the lab. a: Setup, b: Potential and current maintained.

**Cathode (-)**

**Anode (+)**

a

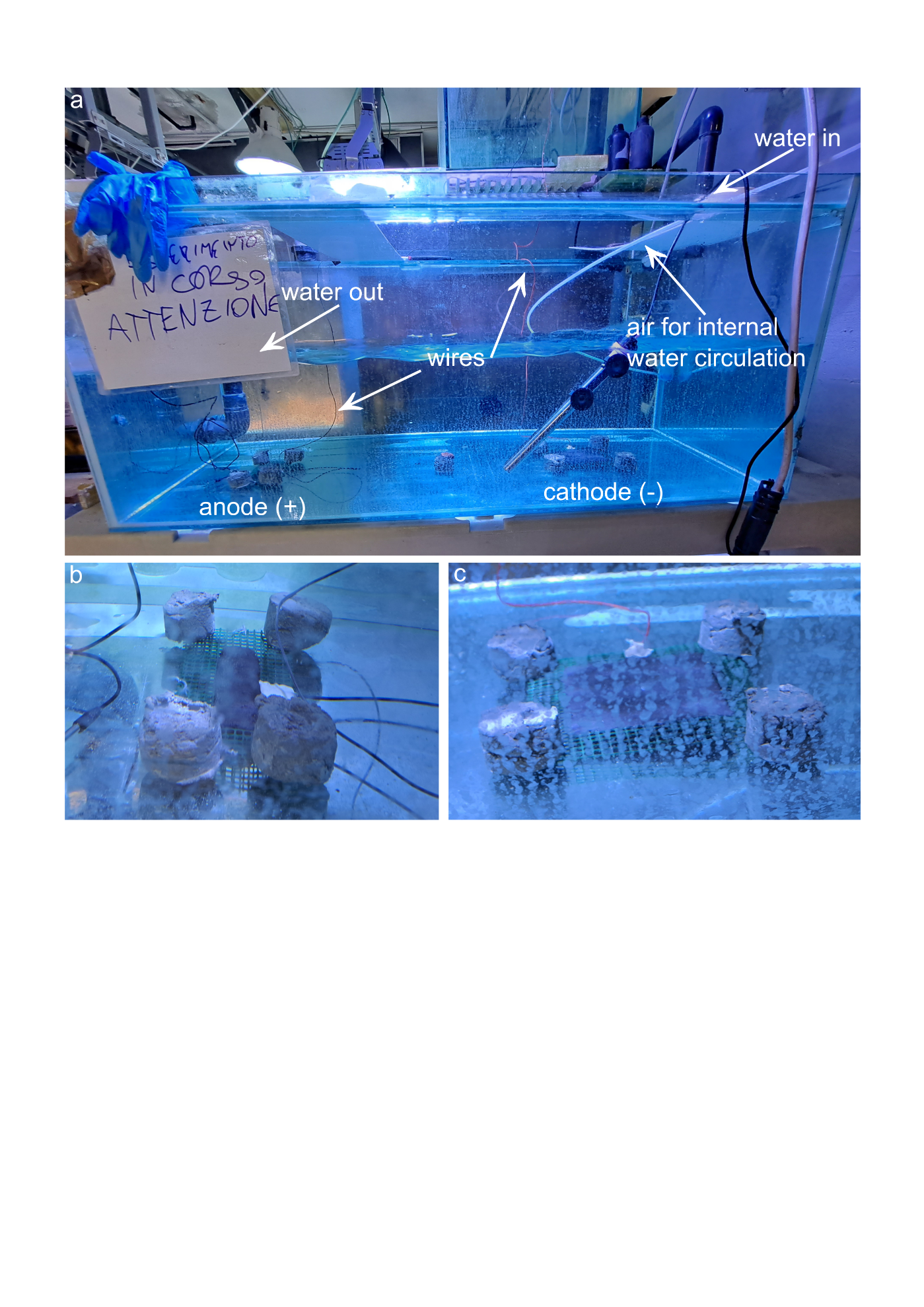


**Figure S 17:** Preliminary setup at Genova Aquarium. a: Whole tank with indication of parts of the experimental setup. b, c: Zoom of the anode (b) and cathode (c).

**Mineral Accretion Technology (MAT) experiments on live corals at Genova Aquarium**

**
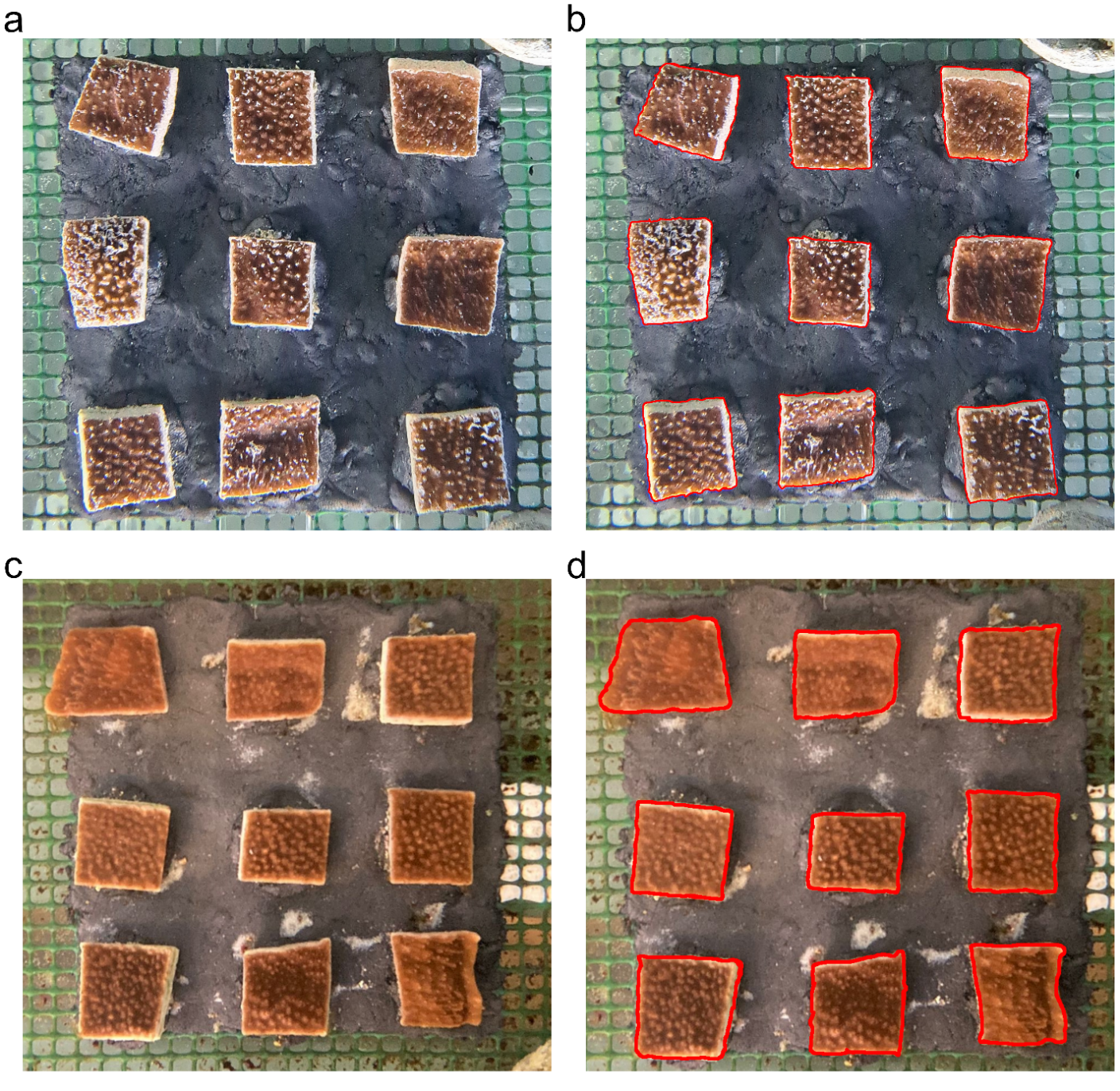
**

**Figure S 18:** Two examples (a-b / c-d) of corals' area selection with ImageJ software.


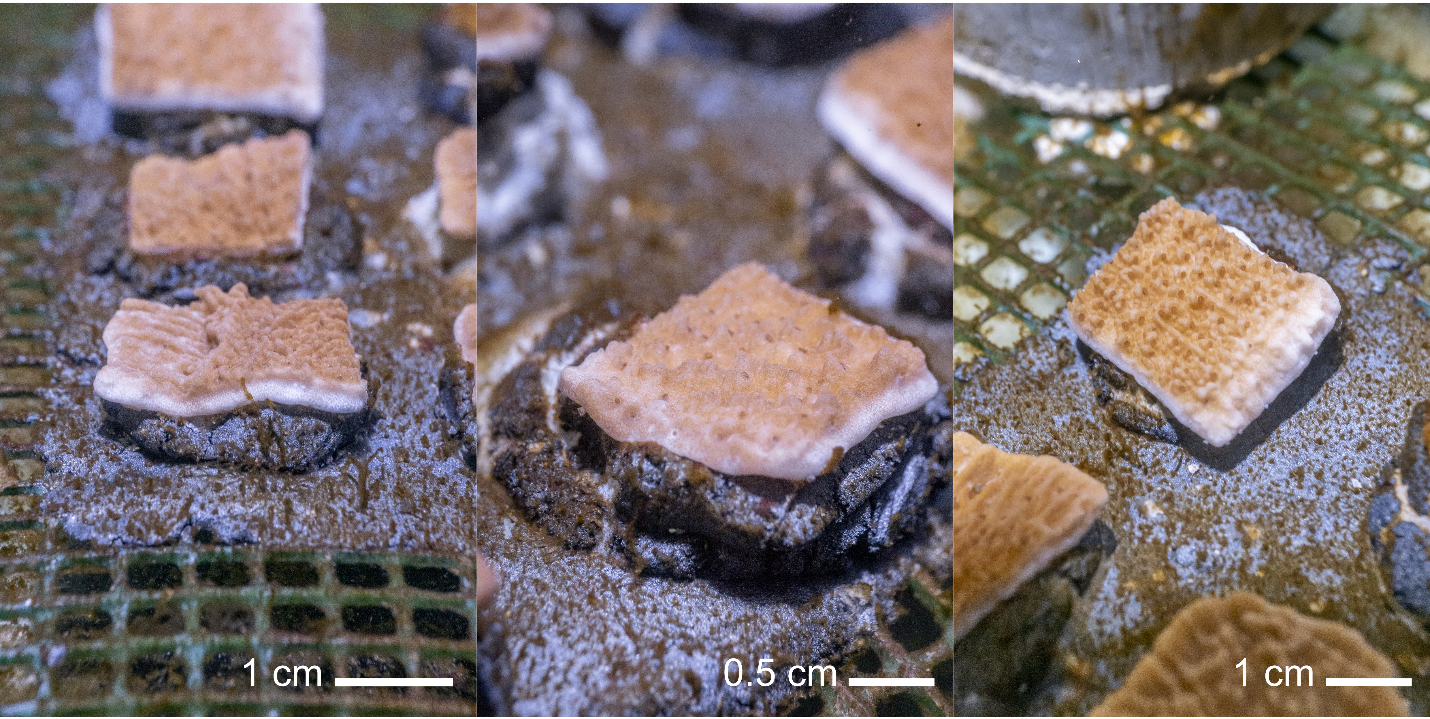


**Figure S 19**: Photos of coral fragments during MAT experiment.


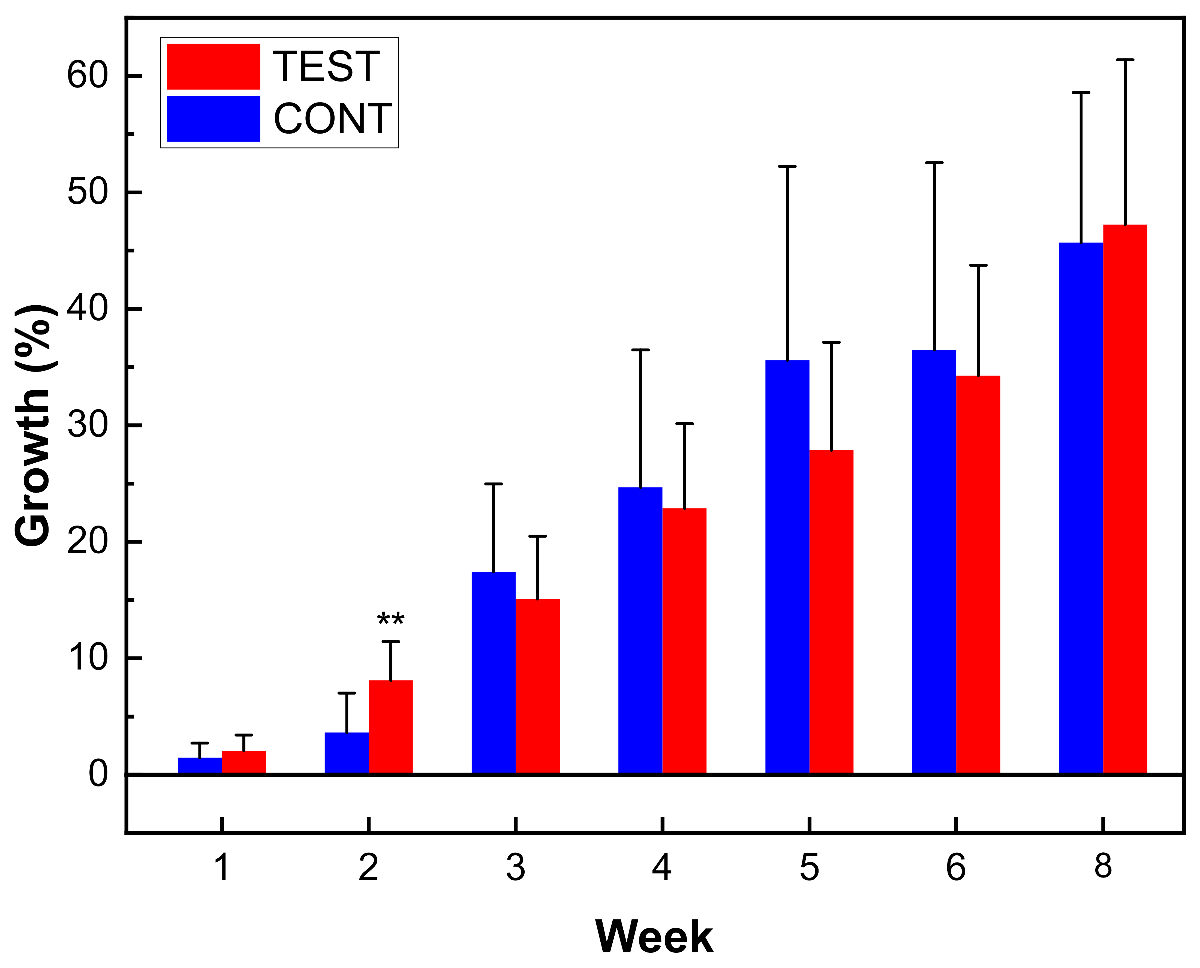


**Figure S 20:** Weekly percentage of growth area (x-y plane) for test and control coral fragments throughout eight weeks.


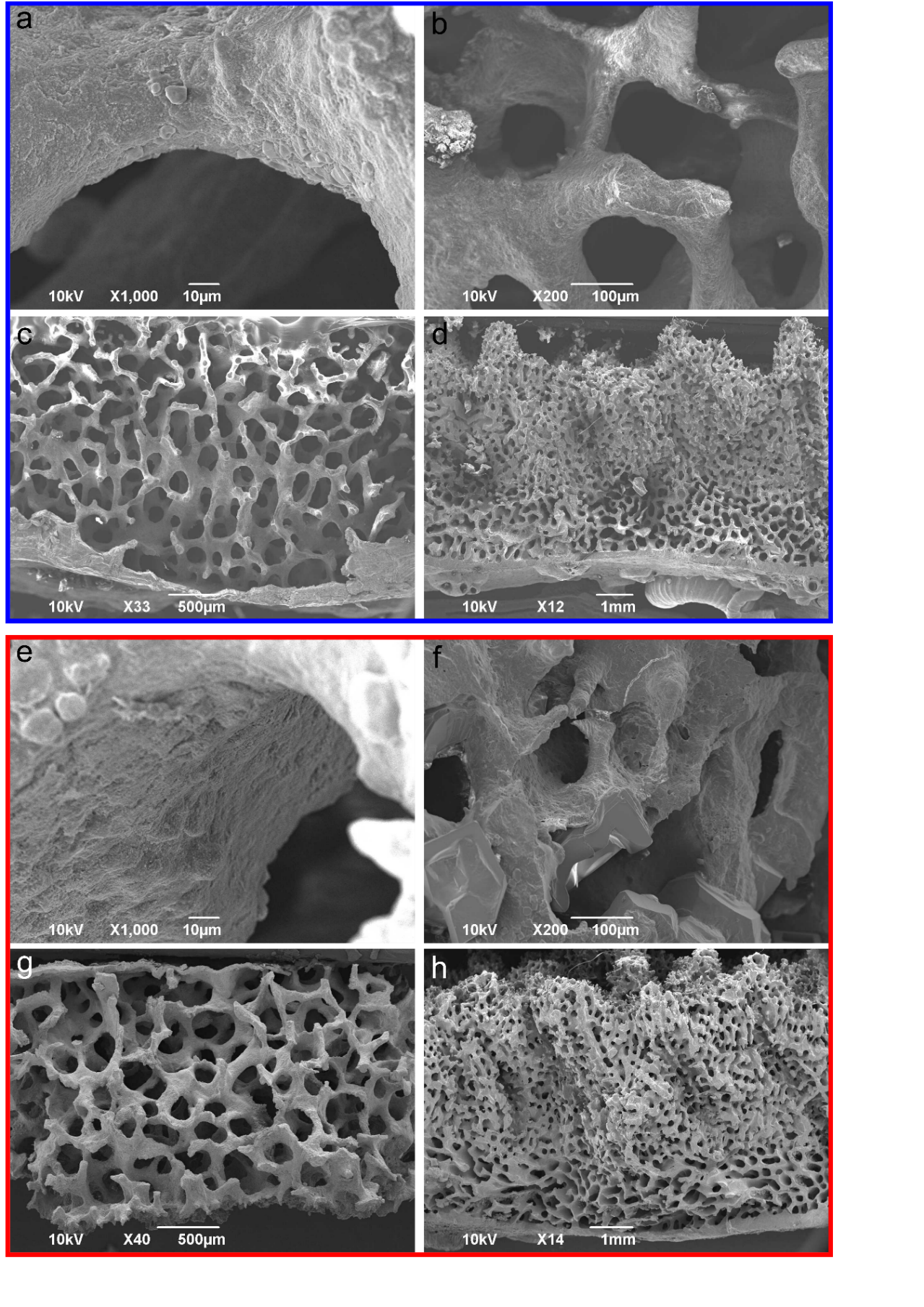


**Figure S 21:** SEM images for the cross-sections of one representative control (a-b-c-d) and test (e-f-g-h) fragment at different magnifications.


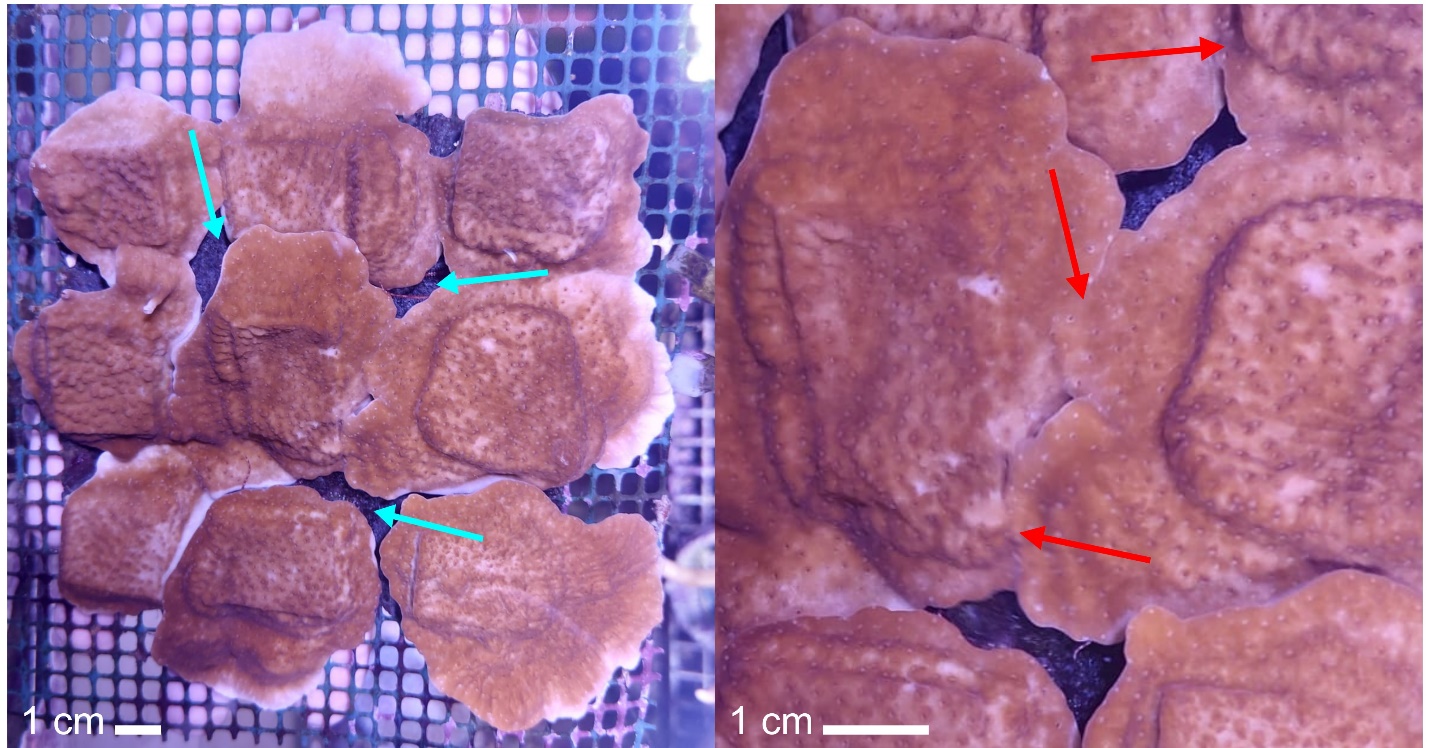


**Figure S 22:** Montipora fragments grown on conductive biopaste 1 year after positioning and > 6 months after the end of MAT experiment. Light blue arrows point to the spots where the conductive biopaste is still visible under the corals. Red arrows indicate fusion points between coral fragments of the same colony.

**Mineral Accretion Technology (MAT) experiments on the field, at MaRHE center (Magodhoo island, Maldives)**

In **Figure S 23,** pictures from an experimental MAT setup at the MaRHE center on Magoodhoo island are shown. **Figure S 23a** displays a scheme of the desired setup: corals are attached with the conductive paste to the sea floor and both electrodes are made with the bicomponent paste. A solar panel anchored to a buoy supplies electricity underwater. This solution grants versatility for the location of the experiment and does not necessitate big metallic structures or proximity to the shore. This experimental setup is designed to allow fast initial growth of the coral fragments while ensuring safe and long-lasting attachment to the reef. Electricity should be supplied for a controlled, finite period to enhance coral growth during the initial stages. Once the corals have established themselves on the paste and become securely anchored to the reef, the electrical supply, solar panel, and wiring should be removed to allow the corals to continue growing autonomously and indefinitely. In this way the only human-made, material left underwater would be the conductive paste, and any potential problems coming from metal oxidation would be totally mitigated. In the remaining panels of **Figure S 23,** pictures of a real MAT experiment done at the MaRHE center are presented. It is interesting to see how both the electrodes' **Figure S 23c, 23d** and **23e** and the attachment, **Figure S 23f,** are optimally realized with the conductive bicomponent.


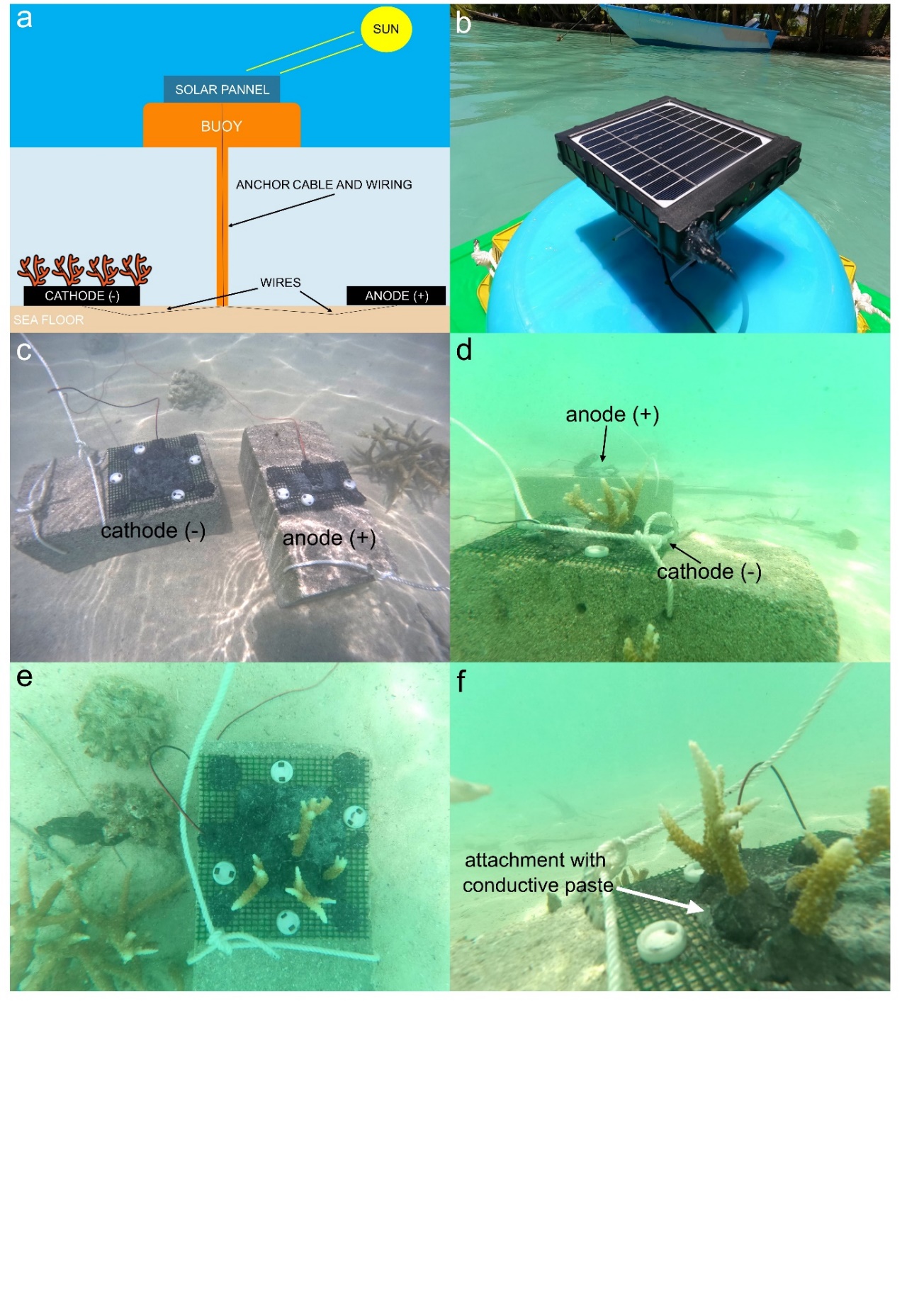


**Figure S 23:** MAT experiment at MaRHE Center on Magoodhoo Island. a: Scheme of the employed setup. b: Solar panel positioned on buoy. c, d: Anode and cathode before and after coral attachment, respectively. e: Zoom from above of the cathode with attached corals. f: Details on the attachment made with conductive bicomponent paste.

**Thermogravimetric analysis**

**Figure S 24** presents the results for the thermogravimetric analysis (TGA) for crosslinked AB pastes with GnPs loading ranging from 10 to 30% by wt. As visible from the graphs, all the samples start to slightly degrade around 150 °C and present a swift degradation between 300 and 400 °C, with the fastest degradation occuring around 370 °C (**Figure S 24b**)*.* Looking at **Figure S 24a**, after 400 °C no further degradation occurs. Examining the residual weight gives confirmation that the portion of the pastes that has been degraded within the temperature range considered is the soybean oil matrix, while the conductive carbon-based filler has not degraded yet. In fact, the residual weight for each sample evaluated coincides with its filler loading mass.


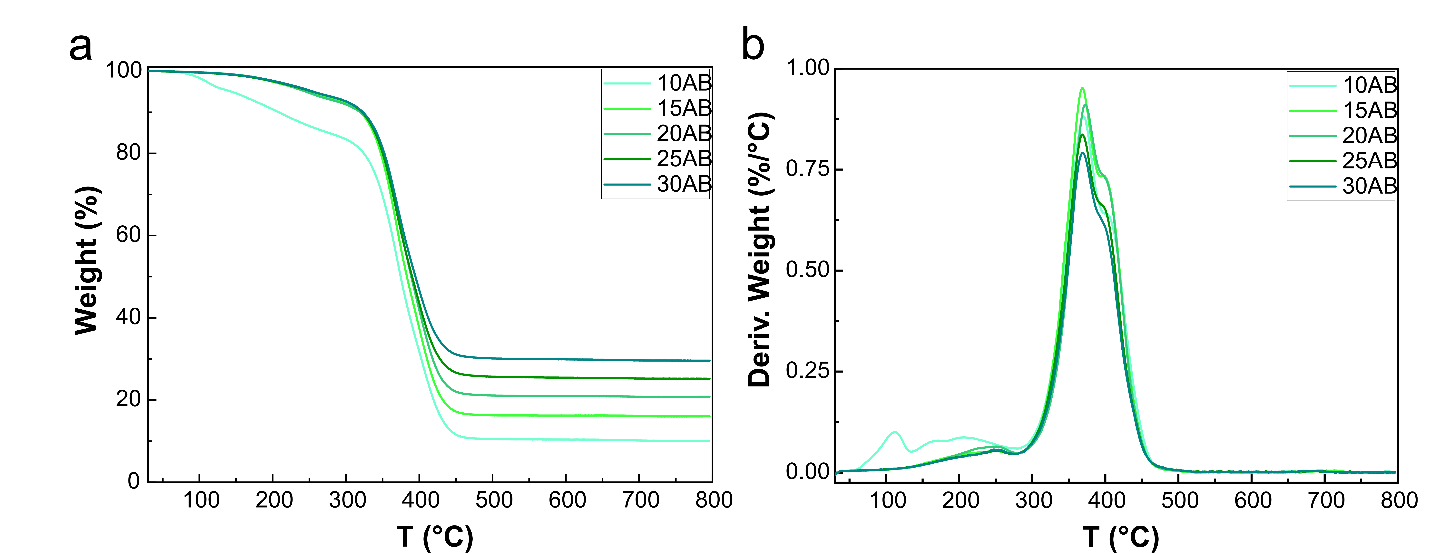


**Figure S 24:** a: Weight percentage and b: derived weight as function of temperature for paste AB with increasing GnPs loading.
